# Extended Directed Fuzzy Social Network Analysis: A framework and application to curriculum networks in Chinese vocational education

**DOI:** 10.1371/journal.pone.0335175

**Published:** 2025-10-27

**Authors:** Borui Zuo, Keqi Shang, Jie Zhang, Manyu Peng, Zhiming Zhu

**Affiliations:** 1 School of Rail Transit, Yunnan Vocational College of Transportation, Kunming, China; 2 School of Intelligent Transportation, Yunnan Vocational College of Transportation, Kunming, China,; 3 Faculty of Transportation Engineering, Kunming University of Science and Technology, Kunming, China; Central South University, CHINA

## Abstract

Due to the differences in node types and the diversity of network relationships, Fuzzy Social Network Analysis (FSNA) needs to specifically address the issues of network heterogeneity and relationship ambiguity. To address this challenge, we propose a new analytical framework called Extended Directed Fuzzy Social Network Analysis Framework (EFDSNAF), which establishes the Typical Connections to assist in evaluating the fuzzy network. Meanwhile, in the area of fuzzy centrality measures, we enhance the variability of the Fuzzy Intensity of Path and propose the term “Total Fuzzy Intensity of Path” (TFIP), considering the distinct characteristics of different networks may lead to variations in path intensity expressions and differences in closeness relationships. Based on this, we optimize the computational methods for fuzzy betweenness centrality and fuzzy closeness centrality, with the efficacy of the method being demonstrated through two examples. Then we applied EDFSNAF to analyze Chinese vocational education curriculum network, with empirical investigation on the Urban Rail Transit Operation and Management Major (URTOMM) and Urban Rail Transit Communication and Signaling Technology Major (URTCSTM). Through EDFSNAF, core courses were identified, and network metrics for different majors effectively captured essential disciplinary differences between the two fields, clearly demonstrating the effectiveness of EDFSNAF.

## 1. Introduction

As a pivotal paradigm for dissecting complex socio-technical systems, Social Network Analysis (SNA) reveals the patterns of information flow and identifies critical nodes within networks through the topological modeling of nodes (individuals, organizations, etc.) and edges [1–3]. With its application scenarios expanding from micro-level interpersonal relationships to macro-level regional networks [4–7] the SNA methodology has undergone comprehensive development,such as: (1) Refinement of network characterization: From unweighted to weighted/directed networks [8–9]; (2) Dynamic modeling: Paradigm shift from static topology to dynamic evolutionary modeling [10]; (3) Increasing Complexity in Relational Cognition: A Cognitive Breakthrough from Deterministic Relations to Fuzzy Relations [11].

Fuzzy Social Network Analysis (FSNA) constructs a mathematical characterization framework for fuzzy-uncertain dual attributes in social networks through the organic integration of SNA and fuzzy set theory. This framework converts real-world uncertainties into computable membership functions, offering advantages for analyzing networks with ambiguous boundaries [12–13].

With the advancement of theoretical research, FSNA has made significant progress in various fields, including network structure analysis [14], node centrality quantification [11,15], and community structure detection [16]. These breakthroughs substantially enhance FSNA’s modeling precision and explainability. Current advancements in FSNA are chiefly manifested in the following areas: (1) Network construction optimization: The fuzzy number-based edge weight representation method [15] preserves uncertainty characteristics in relationships more comprehensively compared to traditional single-value membership approaches. (2) Centrality analysis improvement: By replacing shortest path assumptions with optimal path strategies in fuzzy networks [11,15], novel metrics including fuzzy closeness centrality and fuzzy betweenness centrality have been developed.

With the expansion of FSNA application scenarios and the improvement of analysis accuracy, the inherent heterogeneity of node types and diversity of relational patterns have become increasingly apparent, posing the following critical challenges:

(1) The existing framework fails to fully consider the difference of node types and the fuzziness of the relationship between nodes, which renders the construction of fuzzy networks a significant challenge. The root cause lies in the absence of a universal framework for describing fuzzy relationships. This limitation further restricts the broader application of FSNA methods. (2) The existing frameworks overlook network heterogeneity and differences in propagation patterns. Using homogenized computing paradigm to evaluate node importance (including path calculation rules and influence transmission mechanism) may lead to distortion of information transmission pathways, and then lead to biases in the identification of critical nodes, ultimately hindering the accurate representation of the intrinsic characteristics of real-world interaction networks.

These limitations directly impact the practical application of FSNA methods. To address these fundamental challenges, this study proposes an Extended Directed Fuzzy Social Network Analysis Framework (EDFSNAF). The framework introduces two key innovations: (1) It establishes the concept of “typical connections” to provide a systematic and domain-agnostic approach for defining fuzzy relationships in networks, addressing the long-standing challenge of relationship fuzziness characterization; (2) It develops enhanced fuzzy centrality metrics that account for network heterogeneity by redefining the computational methods for fuzzy betweenness, and closeness centralities, enabling more accurate identification of critical nodes in diverse network contexts.

To demonstrate the framework’s effectiveness and practical applicability, we conduct an empirical analysis using Chinese vocational education curriculum networks as a case study. This application domain presents unique advantages for validation: it incorporates human-factor assessment elements typical of social networks while exhibiting significant node heterogeneity, making it an ideal testbed for evaluating the proposed framework’s capabilities in handling complex fuzzy relationships.

The remainder of this paper is organized as follows: Section 2 provides a comprehensive literature review of FSNA development. Section 3 presents the theoretical foundations underlying our framework. Section 4 details the EDFSNAF methodology, including the typical connection concept and enhanced fuzzy centrality measures. Section 5 demonstrates the framework’s application through case studies of vocational education curriculum networks. Section 6 concludes with discussions on implications and future research directions.

The rest of this paper is structured as follows: Section 2 reviews existing FSNA literature. Section 3 covers theoretical foundations. Section 4 presents our EDFSNAF methodology. Section 5 applies the framework to vocational education curriculum networks. Section 6 concludes with implications and future directions.

## 2. Literature review

The research framework of FSNA primarily comprises two key components: network construction and network analysis. This section begins with a systematic review of research advancements in FSNA network construction, followed by an in-depth discussion on the core issue of network analysis in FSNA: The application and development of centrality measures in FSNA. Finally, it concludes with a summary of the limitations in current research.

### 2.1. Network construction

The network construction paradigm in SNA has evolved from simplified models to complex representations. Classical SNA research [8,16] established undirected and unweighted networks as the fundamental framework, utilizing binary adjacency matrices to characterize the existence of social relationships. While such models can reveal basic inter-node correlations, their neglect of relationship directionality and strength heterogeneity significantly limits their ability to model real-world social interactions [9,17]. In response, the introduction of directed networks [18] and weighted networks [19] marked a significant breakthrough in network modeling, enabling the quantitative characterization of information propagation directionality and interaction strength variability.

However, existing studies reveal that even in weighted networks, a single scalar value remains inadequate for fully characterizing the cognitive fuzziness and evaluative subjectivity inherent in social relationships [15]. This limitation has catalyzed the development of the theoretical framework of FSNA [11–16,20–22]. By incorporating fuzzy set theory, FSNA extends edge weights to membership functions or fuzzy numbers, thereby enabling multidimensional representation of uncertainty in social relationships. Notably, Brunelli and Fedrizzi [20] pioneered the m-ary fuzzy relation model, which generalizes binary {0,1} relationships to the continuous [0,1] interval through membership functions. This paradigm has been extensively applied in subsequent studies [11,16,22] for modeling relational and organizational collaboration networks. It is noteworthy that recent research by Porreca et al [15] identified theoretical limitations in traditional membership degree approaches for characterizing fuzziness, leading to the proposal of a novel edge weight representation method based on fuzzy numbers. This advancement provides new insights for network construction in fuzzy SNA. Edges with fuzzy attributes can more accurately represent relationships between nodes in practice [15]. However, existing research lacks in-depth discussion on how to construct such fuzzy edges and has yet to establish a unified standard. This deficiency may result in arbitrariness in the construction of FSNA networks, impeding the establishment of a consistent FSNA research paradigm across diverse domains and limiting its capacity to meet the growing demand for precise modeling in FSNA. This suggests that future research should focus on addressing the precise expression of relational fuzziness.

### 2.2. Fuzzy centrality measure

In the stage of network analysis, determining centrality metrics is an important research direction for identifying the key nodes in a network [1,23–25]. Based on the concept of communication paths, Bavelas [26] first introduced a centrality measuring approach to explore the relationship between group functionality and network structure. Subsequently, Freeman [27], reviewing multiple existing centrality standards, proposed three key advantages of central nodes compared to other nodes: they have more connections, can reach other nodes more quickly, and control the flow of information between other nodes. Based on these three characteristics, degree centrality, closeness centrality, and betweenness centrality were formally defined and widely promoted. These three centrality measures have been extensively applied across various fields, and further developed in studies on directed networks [18] and weighted networks [19], gradually becoming the most popular centrality metrics [11]. Meanwhile, other centrality methods have also drawn the attention of researchers. For instance, Katz [28] introduced Katz centrality, Bonacich [29] proposed eigenvector centrality, Sohn and Kim [30] developed regional centrality, and Chung [31] introduced Laplacian centrality. Based on the concept of Laplacian centrality, Qi et al [32] further developed a method for calculating Laplacian centrality in weighted networks. Furthermore, Jackson [33,34] proposed decay centrality, which takes into account the attenuation of node influence with increasing distance. Additionally, Shapley centrality [35] has been developed based on hop distances and game-theoretic principles.

With increasing attention to fuzziness in network construction, centrality metrics in SNA have been extended to fuzzy centrality measures [5,11]. Brunelli and Fedrizzi [20] pioneered the integration of fuzzy membership functions with traditional centrality theory, and established a foundational framework for fuzzy centrality indices, laying the theoretical foundation for future research. Wang and Gong [13] further advanced this field by combining fuzzy theory with hypergraph theory to develop centrality measures based on fuzzy hypergraphs. Liao et al [36] redefined fuzzy degree centrality and fuzzy closeness centrality through analyzing the operational forms of fuzzy relationships. Considering the difference of node types and the multiple relationships between nodes. Lu et al [31] proposed a comprehensive set of metrics, including fuzzy node degree, fuzzy cohesion degree, fuzzy clustering coefficient, and fuzzy geographical concentration, which were validated across three distinct fuzzy networks: friend groups, forum communities, and relational networks. However, their study did not conduct an in-depth analysis of the applicability of identical fuzzy centrality measures across different networks. Hu and Zhang [14] introduced structural hole concepts in directed FSN research. Building upon this, Hu et al [11] developed the concepts of fuzzy degree centrality and fuzzy closeness centrality, along with a computational method for fuzzy connected intensity between nodes. Porreca et al. [15] extended path strength from scalar membership degrees to triangular fuzzy numbers and introduced the “best path” concept based on Hu et al’s [11] fuzzy connected intensity, proposing three triangular fuzzy number-based centrality measures: fuzzy degree centrality, fuzzy closeness centrality, and fuzzy betweenness intensity. However, a common issue in existing FSNA research is the assumption of network homogeneity. Even when modeling methods that retain more real-world information are employed, the rigid centrality calculation framework still fails to accurately capture the heterogeneity of real networks, resulting in significant limitations in key nodes identification.

### 2.3. The conclusions

Based on a comprehensive review of existing literature, the core issues that urgently need to be addressed in FSNA primarily focus on the fuzziness of relationships and the accurate characterization of network heterogeneity.

(1) **Fuzziness of Relationships**: Relationship assessment forms the foundation of network construction, and FSNA emphasizes the fuzziness of relationships, which essentially reflects the need for a more accurate representation of the real world [11,15]. In unweighted networks, relationship construction is relatively simple, typically judged from a binary perspective to determine whether a connection exists. In weighted networks, although the evaluation process is more complex, it can still be measured with a reasonable degree of accuracy using tools such as scales [19]. However, in FSNA, due to the fuzziness of relationships, the traditional “either-or” judgment is no longer applicable. As fuzziness increases, relationship evaluation becomes increasingly complex. Existing studies assume that evaluators can clearly define relationships, and that fuzzy semantics provided through subjective experience can effectively measure fuzzy relationships [15]. But considering the fuzziness of the real world, not all fuzzy relationships can be easily mapped to predefined semantic levels, especially when the relationship being evaluated is not interpersonal. Moreover, the same semantic expression may carry different meanings in different contexts or for different evaluators, leading to biased evaluation results [37]. Therefore, during the network construction phase, establishing an effective framework for describing fuzzy relationships is crucial for enhancing the accuracy of the network.
(2) **Network Heterogeneity Characteristics**: Variations in node types and relational attributes lead to pronounced network heterogeneity. In the network construction stage, such heterogeneity is exhibited as disparities in the metrics of connectivity relationships, while in centrality analysis, it reflects the differentiated requirements for computational methods under the same evaluation objectives [19]. Current centrality metrics generally overlook potential impacts of network heterogeneity, applying uniform computational methods across diverse networks (For example, Porreca et al [15]). While such simplified models help highlight network commonalities and suit general SNA scenarios, they risk information distortion and inadequate reflection of actual interaction network characteristics. Given FSNA’s core objective of authentic real-world representation, incorporating network heterogeneity in fuzzy centrality metric construction is essential. Adaptive parameter adjustments for fuzzy centrality metrics with identical semantics, tailored to specific network structural features, can more accurately simulate network structures and reflect node significance.

Addressing these research limitations, this study proposes an Extended Directed Fuzzy Social Network Analysis Framework (EDFSNAF), aiming to enhance existing FSNA models and improve their applicability across diverse node types and network relationships.

## 3. Preliminaries

This section delineates the theoretical underpinnings of the EDFSNAF we proposed. First, introduces the basic network model of FSN that serves as the basis for subsequent extensions. Second, revisits the conceptual evolution of three centrality metrics – degree centrality, closeness centrality, and betweenness centrality systematically. Finally, introduces the fuzzy set theoretical framework and essential arithmetic operations (triangular fuzzy number addition, multiplication, and defuzzification) to underpin EDFSNAF’s computational architecture.

### 3.1. Fundamentals of FSNA

A Social Network (SN) can be represented as G={V,E,W} ,where: $V=v_{1},v_{2},...,v_{n}$, represents the nodes in the network; $E=\{e_{ij};i,j\in V\}$ denotes the edges in the network; $W=\left\{w_{ij};i,j\in V\right\}$ represents the mutual influence degrees of the edges in the network. In directed networks, $e_{ij}\neq e_{ji}$$w_{ij}\neq 1$.

FSNA is developed based on weighted SNA. In FSNA, both nodes and edges can be fuzzy. However, this paper focuses on the case where nodes are explicit and edges are fuzzy, which aligns with most real-world scenarios we are familiar with [11,15]. In FSNA, the network can be described as:

$G_{f}=\left(V,\tilde{A}\right)$, where:$V=\left\{v_{1},v_{2},...,v_{n}\right\}$ is the set of explicit nodes; $\tilde{A}=\left(\begin{array}{ccc} {}*20c{\tilde{A}}_{11} & \cdots & {\tilde{A}}_{1n}\\ \vdots & \ddots & \vdots \\ {\tilde{A}}_{n1} & \cdots & {\tilde{A}}_{nn} \end{array}\right)$ is the fuzzy adjacency matrix corresponding to nodes V, where ${\tilde{A}}_{ij}$ represents the fuzzy relationship between node *i* and node *j*. In directed networks, it is typically the case that ${\tilde{A}}_{ij}\neq {\tilde{A}}_{ji}$.

### 3.2. Introduction to centrality indices

Centrality measures have always been a core focus in social network research. These measures help identify key nodes within a network: revealing which nodes occupy central positions, which play leadership roles, which act as intermediaries, and which, if they fail, could have the greatest impact on the overall function of the network. In previous studies, the most important calculation methods undoubtedly include degree centrality, closeness centrality, and betweenness centrality [11].

#### 3.2.1. Degree centrality.

Degree centrality reflects the extent to which a node is directly popular or the number of its direct connections with other nodes [23].

(1) In a classical unweighted and undirected network, a node degree refers to the number of edges connected to it. The specific expression is:

$$C_{d}\left(v_{i}\right)=\sum_{j,j\neq i}d_{ij}$$ (1)

where: $C_{d}\left(v_{i}\right)$ represents the degree centrality of node $v_{i}$; $d_{ij}$ is an element in the adjacency matrix, where $d_{ij}=1$ if there is an edge between node *v*_*i*_ and the node *v*_*j,*_ and $d_{ij}=0$ otherwise;

(2) In directed and unweighted networks, connections between nodes are directional. Therefore, the degree of a node can be further divided into Out-Degree $C_{d}^{out}\left(v_{i}\right)$, In-Degree $C_{d}^{in}\left(v_{i}\right)$, and Total-Degree $C_{d}^{total}\left(v_{i}\right)$, defined as follows:

$$C_{d}^{in}\left(v_{i}\right)=\sum_{j,j\neq i}d_{ij}^{in}$$ (2)

$$C_{d}^{out}\left(v_{i}\right)=\sum_{j,j\neq i}d_{ij}^{out}$$ (3)

$$C_{d}^{total}\left(v_{i}\right)=C_{d}^{in}\left(v_{i}\right)+C_{d}^{out}\left(v_{i}\right)$$ (4)

In-degree centrality indicates the popularity of a node within the network, while out-degree centrality measures the gregariousness of a node. Specifically: If there is a direct connection from node *j* to node *i*, $d_{ij}^{in}=1$; If there is a direct connection from node *i* to node *j*, then $d_{ij}^{out}=1$.

(3) In FSNA, Hu et al [11] proposed a calculation method for fuzzy degree centrality. Taking fuzzy in-degree centrality as an example, it is expressed as:

$${\tilde{d}}_{I}\left(v_{i}\right)=\sum_{j=1,j\neq i}^{n}{\tilde{A}}_{ji}$$ (5)

Where: ${\tilde{d}}_{I}\left(v_{i}\right)$ represents the fuzzy in-degree centrality. ${\tilde{A}}_{ij}$ represents the fuzzy relationship from node *j* to node *i*.

#### 3.2.2. Closeness centrality.

Closeness Centrality is a key metric that measures how “close” a node is to all other nodes in the networks. It reflects the average distance from a node to all other nodes, with higher closeness centrality indicating greater efficiency in information propagation within the network.

(1) In an unweighted network, the calculation method for closeness centrality is as follows:

$$C_{c}\left(v_{i}\right)=\frac{\text{1}}{\frac{\text{1}}{n-1}\sum_{v_{j}\neq v_{i}}l_{ij}}$$ (6)

Where: $C_{c}\left(v_{i}\right)$ denotes the closeness centrality of node *v*_*i*_; $l_{ij}$ represents the shortest path length between node *v*_*i*_ and node *v*_*j*_;.

(2) In fuzzy networks, closeness centrality indices are calculated based on the strength of fuzzy relationships. Hu et al [11] introduced the concept of Fuzzy Intensity of Path and Fuzzy Connected Intensity.

**Definition 3.1 Fuzzy Intensity of Path**.Suppose there exists a path $v_{0}\cdot {\tilde{A}}_{1}\cdot v_{1}\cdot {\tilde{A}}_{2}\cdot v_{2}...{\tilde{A}}_{k}\cdot v_{k}$*.* $\left\{{\tilde{A}}_{1},{\tilde{A}}_{2},...,{\tilde{A}}_{k}\right\}$ is a sequence of edges from *v*_*0*_ to *v*_*k。*_The fuzzy intensity of path *w*, denoted as ${\tilde{s}}_{d}\left(\tilde{w}\right)$.


$${\tilde{s}}_{d}\left(\tilde{w}\right)=\underset{i=1}{\overset{k}{\wedge }}\mu \left({\tilde{A}}_{i}\right)$$ (7)

Where ${\tilde{s}}_{d}\left(\tilde{w}\right)$ is calculated as the minimum membership function value among all edges in the path; $\mu \left({\tilde{A}}_{i}\right)$ is a membership function of ${\tilde{A}}_{i}$。

**Definition 3.2 Fuzzy Connected Intensity**.If there are *n* paths from *u* to *v* in *G*_*f*_, then $\tilde{s}\left(u,v\right)$ is called a fuzzy connected intensity from *u* to *v*, which is calculated as the maximum membership function value of the best path among all paths.


$$\tilde{s}\left(u,v\right)=\underset{k=1}{\overset{n}{\vee }}{\tilde{s}}_{d}\left({\tilde{w}}_{k}\right)$$ (8)

Subsequently, take fuzzy in-closeness centrality as example, it can be expressed as ${\tilde{C}}_{CI}\left(v_{i}\right)$:


$${\tilde{C}}_{CI}\left(v_{i}\right)=\sum_{j=1,j\neq i}^{n}\tilde{s}\left(v_{i},v_{j}\right)$$ (9)

#### 3.2.3. Betweenness centrality.

Betweenness centrality measures the ability of a node to act as a “mediator” or “bridge.” Specifically, it quantifies the frequency with which a node is included in the shortest paths between all pairs of nodes in the network. A higher betweenness centrality indicates that the node plays a more significant bridging role in the network, exerting greater influence on the transmission of information or resources.

(1) Betweenness Centrality in Unweighted Networks

Whether in directed or undirected networks, the method for calculating betweenness centrality in unweighted networks remains consistent. The formula is as follows:


$$C_{b}\left(v_{i}\right)=\sum_{s\neq v_{i}\neq t}\frac{\sigma_{st}\left(v_{i}\right)}{\sigma_{st}}$$ (10)

Where $C_{b}\left(v_{i}\right)$ denotes the betweenness centrality of node *v*_*i*;_ $\sigma_{st}$ represents the total number of shortest paths between node *s* and node *t*; $\sigma_{st}\left(v_{i}\right)$ represents the number of shortest paths between node *s* and node *t t*hat pass through node *v*_*i*_.

(2) Betweenness Centrality in Fuzzy Networks

Since the work of Hu et al [11] does not involve intermediary centrality, we take Porreca et al [15] as an example. The intermediary centrality in fuzzy networks is defined as follows:


$$B\left(v_{i}\right)=\sum_{s\neq t\neq v_{i}}\frac{P_{st}\left(v_{i}\right)}{P_{st}}$$ (11)

$B\left(v_{i}\right)$ is the fuzzy betweenness centrality. where $P_{st}\left(v_{i}\right)$ indicate the number of $\tilde{s}\left(s,t\right)$ between *s* and *t* that passes thought *i*, and $P_{st}$ is the total number of $\tilde{s}\left(s,t\right)$ between node *s* and node *t*.

### 3.3. Definition of Triangular Fuzzy Numbers (TFN) and basic arithmetic operations

For detailed information on fuzzy theory, refer to [38–39]. Additional extensions of fuzzy computational frameworks and improvements in computational efficiency are discussed in [40–42]. This section primarily introduces the fuzzy mathematical operations used later in this paper.

#### 3.3.1. Triangular Fuzzy Numbers (TFN).

A fuzzy set can be defined as: $A=\left\{\left(x,\mu_{A}\left(x\right)|x\in A,\mu_{A}\left(x\right)\in \left[0,1\right]\right)\right\}$, where $\mu_{A}\left(x\right)$ is the membership function, which represents the degree to which a parameter *x* belongs to the fuzzy set A. Among various types of fuzzy numbers, TFN have garnered widespread attention due to their simplicity and intuitiveness.

**Fig 1:**
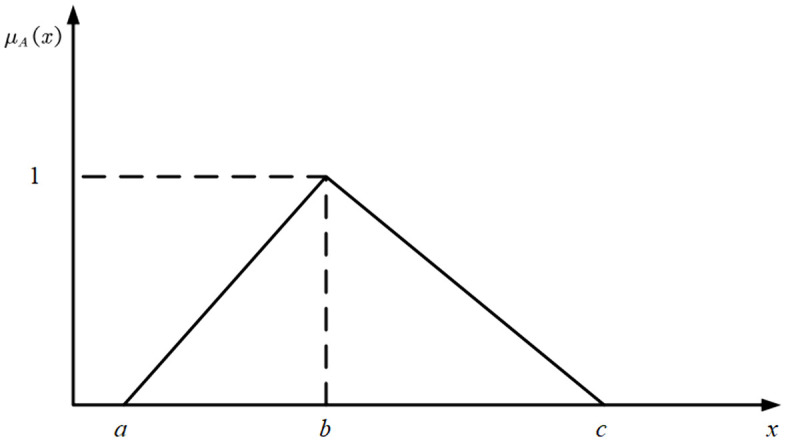
Triangular fuzzy number (TFN).

In the TFN as shown in Fig 1, a fuzzy set *A* can be represented as *[a,b,c]*, where *0 < a < b < c*. The specific definitions are as follows:

(1) a represents the left end point, c represents the right end point, and b is the peak value;
(2) When *x < a or x > c*, $\mu_{A}\left(x\right)=0$, indicating that *x* does not belong to the fuzzy set;
(3) when x = *b*, $\mu_{A}\left(x\right)=1$, indicating that x fully belongs to the fuzzy set. Within the intervals [*a,b*] and [*b,c*], the membership function $\mu_{A}\left(x\right)$ is expressed as:

$$\mu_{A}\left(x\right)=\{\begin{array}{c} {}*20l\frac{x-a}{b-a}\;\;\;\;\;\;\;\;\;\;\;\;\;\;\;\;\;\;\;\;\;\;\;\;\;\;a\leq x\leq b\;\;\\ \frac{c-x}{c-b}\;\;\;\;\;\;\;\;\;\;\;\;\;\;\;\;\;\;\;\;\;\;\;\;\;\;b\leq x\leq c\;\; \end{array}$$ (12)

In FSNA, the fuzzy edge ${\tilde{A}}_{ij}$ can be represented as a TFN in the form $\left[a_{ij},b_{ij},c_{ij}\right]$. where: For undirected networks, ${\tilde{A}}_{ij}={\tilde{A}}_{ji}$ must hold; For directed networks, ${\tilde{A}}_{ij}\;\;and\;\;{\tilde{A}}_{ji}$ are not necessarily equal.

#### 3.3.2. Basic arithmetic operations of TFN.

Suppose there are *n* triangular fuzzy numbers ${\tilde{A}}_{n}=\left[a_{n},b_{n},c_{n}\right]$, their operations can be expressed as follows:

(1) Addition

$${\tilde{A}}_{1}⊕{\tilde{A}}_{2}=\left[a_{1}+a_{2},b_{1}+b_{2},c_{1}+c_{2}\right]$$ (13)

(2) Multiplication

$${\tilde{A}}_{1}⊗{\tilde{A}}_{2}=\left[a_{1}\times a_{2},b_{1}\times b_{2},c_{1}\times c_{2}\right]$$ (14)

(3) Scalar Multiplication

$$q⊙{\tilde{A}}_{1}=\left[q\times a_{1},q\times b_{1},q\times c_{1}\right]$$ (15)

(4) Fuzzy triangular aggregation operator [39]

$$\underset{i=1}{\overset{n}{⊕}}Q⊙{\tilde{A}}_{i}=\left[\sum_{i=1}^{n}q_{i}\times a_{i},\sum_{i=1}^{n}\begin{array}{c} {}*20cq_{i}\times b_{i},\sum_{i=1}^{n}q_{i}\times c_{i} \end{array}\right]$$ (16)

(5) Fuzzy triangular ordered aggregation operator

$$\underset{i=1}{\overset{n}{⊕}}Q⊙{\tilde{A}}_{i}^{e}=\left[\sum_{i=1}^{n}q_{i}\times a_{i}^{e},\sum_{i=1}^{n}\begin{array}{c} {}*20cq_{i}\times b_{i}^{e},\sum_{i=1}^{n}q_{i}\times c_{i}^{e} \end{array}\right]$$ (17)

where $Q=\left[q_{1},q_{2},...,q_{n}\right]$ is the tuning parameter of the fuzzy set $\tilde{A}$, ${\tilde{A}}_{i}^{e}$ represents the *i-th* group of ${\tilde{A}}_{i}$ arranged in desscending order based on the centroid method. The triangular fuzzy numbers $\left[a_{i}^{e},b_{i}^{e},c_{i}^{e}\right]$ denotes the fuzzy value of ${\tilde{A}}_{i}^{e}$.

## 4. Methods

This section presents the Extended Directed Fuzzy Social Network Analysis Framework (EDFSNAF), a comprehensive methodological approach developed to address the challenges of relationship fuzziness and network heterogeneity in fuzzy social network analysis. The framework consists of two main components: network construction methodology (Section 4.1) and fuzzy centrality evaluation metrics (Section 4.2).

### 4.1. Construction of the EDFSNAF network

#### 4.1.1. Determination of node evaluation and evaluation scale.

Compared to Undirected Graphs, Directed Graphs can more accurately reflect real-world interactions. Assuming there are *N* nodes participating in the Extended Directed Fuzzy Social Network (EDFSN), the evaluation scale needs to be determined based on the mutual connection relationships between nodes. The determination of the scale is based on past evaluation experiences and expert consensus [15,43]. In this study, the interval *[0,K]* is defined as the evaluation scale for node relationships, representing the strength of the association between nodes.

#### 4.1.2. Determination of typical connections.

Currently, there is no universally applicable FSNA connection establishment paradigm. Existing studies generally follow the process outlined below: First, the model builder defines fuzzy semantics (such as “never,” “occasionally,” and “constantly”) and their corresponding fuzzy values, which are then directly applied for the evaluation between nodes. However, this approach has significant limitations. It largely depends on the subjective experience of the model builder and lacks communication with actual evaluators, leading to the following two issues: (1) **Subjective perception differences**: Different evaluators may interpret the same fuzzy semantic values differently, resulting in biased outcomes; (2) **Insufficient domain adaptability**: With the widespread application of fuzzy networks, the relationships between nodes in many domains are not suitable for differentiation using simple semantic values without explanation.

To overcome these issues, a direction worthy of attempt is fostering collaboration between evaluators and evaluation publishers. Through clear conceptual definitions, fuzzy degree concepts tailored to specific domains can be proposed. Based on this approach, this paper generalizes the steps for connection establishment and presents a simple, universal method for connection establishment based on typical connections.

**Definition 4.1. Typical Connections**

Typical connections reflect the degree distinctions between nodes in a fuzzy network. The essential characteristics of typical connections include:1. Clear fuzzy numbers;2. The ability to provide conceptual differentiation and degree anchoring. In some studies, although the subjective semantic distinctions provided are not systematically defined, they possess clear fuzzy numbers and effectively serve as typical connections. For example, the terms {“never”, “occasionally”, “constantly”} [15] can be regarded as a typical form of connection. In other specific networks, the terms {“no dependency”, “low dependency”, “high dependency”} [43] can similarly be considered typical connections.

**Steps for Defining Typical Connections**

(1) Formation of the preliminary set of typical connections

The evaluators and evaluation publishers, using the Delphi method or other decision-making approaches, discuss the degree of relational strength between the edges in the fuzzy network. By examining these relationships in a manner that moves from distant to close or vice versa, the typical connection relationships within the edge relationships are identified and a consensus is reached. Ultimately, M types of typical connection relationships are established and are preliminarily labeled as TYPE-A to TYPE-M.

(2) Detailed Description and Refinement

Provide a detailed description of the initially formed typical connections and give corresponding examples. Based on this, modify and refine those typical connections that are conceptually unclear or interfere significantly with each other. The finalized typical connection set is summarized in Table 1.

**Table 1:** Typical connection description.

Typical connection	Explanation of the connection	Illustrations of typical connections
TYPE-M (Brief summary of connections)	Clearly describe the relationship between the connections	Determine representative examples of the connection

**Fig 2:**
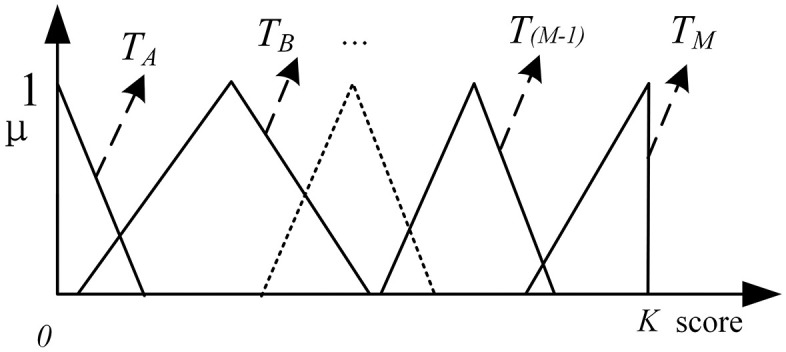
Fuzzy set of typical connections.

(3) Assigning Fuzzy Numbers to Typical Connections. Taking the TFN used in this paper as an example: For each typical connection from TYPE-A (TA) to TYPE-M (TM), the maximum possible point (peak value,which means μ(x)=1) for each typical connection is first preliminarily determined within the evaluation interval [0, K], as shown in Fig 2. This point serves as the core reference value for forming the TFN in subsequent steps. Based on further discussions and reaching a consensus, a complete TFN in the form of [*a, b, c*] is assigned to each typical connection, where [*a*] represents the left endpoint, [*b*] is the peak value, and [*c*] represents the right endpoint. Finally, the fuzzy set for the typical connections is obtained, which is used for the subsequent analysis and modeling of the fuzzy network.

Defining typical connections preferentially not only facilitates the anchoring of a large number of connection evaluations in fuzzy networks, but also plays a critical role in clarifying fuzzy networks into corresponding heterogeneous networks. The subsequent case in this paper demonstrates the application of typical connections in the method of representing connection relationships through fuzzy numbers. In another approach that represents connection relationships through membership degrees(for example, Hu et al [11]), the establishment of typical connections can be regarded as the process of constructing an evaluation fuzzy set.

It should be further emphasized that the approach for establishing typical connections is more akin to a conceptual framework, with the primary goal being to define fuzzy relationships as clearly and completely as possible. The usage of typical connections will be further elaborated in the specific case studies presented later in this paper.

#### 4.1.3. EDFSNAF network construction.

To retain as much valid information as possible, the evaluation content in EDFSNAF can be divided into three categories: typical connections, rigid evaluations, and free fuzzy evaluations. Among them:

(1) **Typical Connections**: Use the fuzzy values of the previously established connections;
(2) **Rigid Evaluations**: Consider cases where evaluators have a clear, non-fuzzy tendency regarding certain relationships;
(3) **Free Fuzzy Evaluations**: Based on typical connections, flexibly determine the fuzzy values of corresponding relationships.

The constructed EDFSN is represented as $G=\left\{V,\tilde{A}\right\}$, where $V=\left\{v_{1},v_{2}....v_{n}\right\}$ denotes the set of nodes:


$$\tilde{A}=\left(\begin{array}{ccc} {}*20c{\tilde{A}}_{11} & \cdots & {\tilde{A}}_{1n}\\ \vdots & \ddots & \vdots \\ {\tilde{A}}_{n1} & \cdots & {\tilde{A}}_{nn} \end{array}\right)$$

is the fuzzy adjacency matrix corresponding to nodes.

Where ${\tilde{A}}_{ij}=\left[a_{ij},b_{ij},c_{ij}\right]$ represents the influence relationship from node *i* to node *j*. For rigid evaluations, to facilitate subsequent centrality calculations, they can be converted into fuzzy modules as follows: $a_{ij}=\left[a_{ij},a_{ij},a_{ij}\right]$.

### 4.2. Fuzzy centrality evaluation

After constructing the fuzzy network, quantitative analysis of the network is required. In the EDFSN, given the limitations of existing fuzzy centrality calculation methods, this section will propose improvements to the current fuzzy centrality metrics to more accurately evaluate the importance of nodes and their roles within the fuzzy network.

#### 4.2.1. Fuzzy degree centrality.

Degree centrality measures the strength of direct connections between a node and other nodes. In fuzzy degree centrality, as the edges in the network are represented by fuzzy numbers, the calculation of fuzzy degree centrality is also based on fuzzy numbers. Fuzzy degree centrality can be further divided into fuzzy in-degree centrality and fuzzy out-degree centrality:

**Fuzzy In-degree Centrality**: Reflects the extent to which a node is influenced by other nodes;

**Fuzzy Out-degree Centrality**: Reflects the extent to which a node exerts influence on other nodes.

To standardize the description and enable comparisons across different networks, the fuzzy in-degree and out-degree numbers need to be normalized to the interval [0,1]. The specific calculation is as follows:


$${\tilde{B}}_{ij}=\left[\frac{a_{ij}}{K},\frac{b_{ij}}{K},\frac{c_{ij}}{K}\right]=\left[{a^{\prime }}_{ij},{b^{\prime }}_{ij},{c^{\prime }}_{ij}\right]$$ (18)

$${\tilde{C}}_{d}^{in}\left(v_{i}\right)=\underset{j\in N,j\neq i}{⊕}\left(q_{j}^{a}⊙{\tilde{B}}_{ji}\right)$$ (19)

$${\tilde{C}}_{d}^{out}\left(v_{i}\right)=\underset{j\in N,j\neq i}{⊕}\left(q_{j}^{a}⊙{\tilde{B}}_{ij}\right)$$ (20)

$${\tilde{C}}_{d}^{tot}\left(v_{i}\right)={\tilde{C}}_{d}^{in}\left(v_{i}\right)+{\tilde{C}}_{d}^{out}\left(v_{i}\right)$$ (21)

Where ${\tilde{B}}_{ij}$ represents the fuzzy influence number of node *i* on node *j*, mapped to the interval [0,1]. ${\tilde{C}}_{d}^{in}\left(v_{i}\right)$,${\tilde{C}}_{d}^{out}\left(v_{i}\right)$, ${\tilde{C}}_{d}^{tot}\left(v_{i}\right)$ denote the fuzzy in-degree, fuzzy out-degree and fuzzy total-degree of node *i*. $Q_{i}^{a}=\left[q_{1}^{a},q_{2}^{a},...,q_{n}^{a}\right]$ is the adjustment coefficient, which aims to normalize the fuzzy influence degrees between different nodes. To unify the scale and facilitate comparisons across different networks, it is suggested that $q_{j}^{a}$ be set as $\frac{\text{1}}{n-1}$.

#### 4.2.2. Fuzzy betweenness centrality.

(1) Limitations of Existing Research on Fuzzy Betweenness Centrality

**Fig 3:**
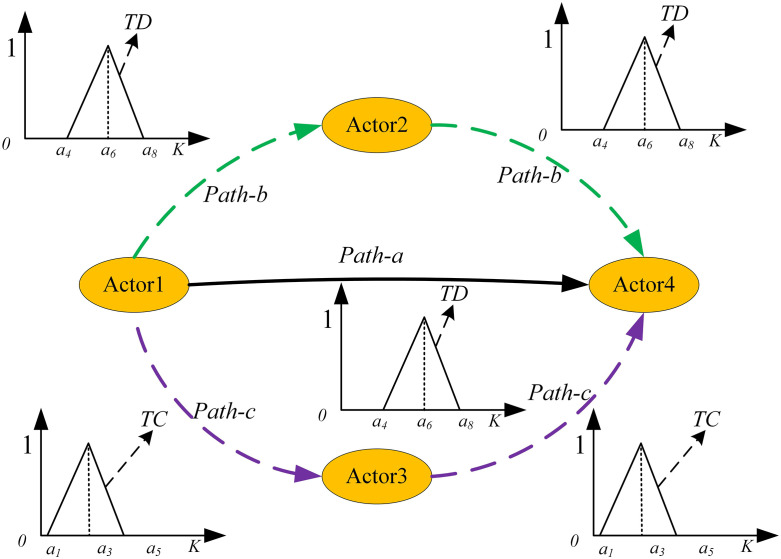
A network with 4 nodes and 3 fuzzy path.

Betweenness centrality, as a core metric for measuring the “bridging” role of nodes in networks, occupies a prominent position in classical SNA. Traditional methods typically calculate betweenness centrality based on shortest paths, which performs well in unweighted deterministic networks. However, when confronted with the inherent uncertainty and fuzziness of connection strengths in fuzzy social networks, traditional approaches reveal significant inadequacies. While Porreca et al. (2025) [15] pioneered a computational framework for fuzzy betweenness centrality based on the “best path” concept, establishing an important foundation for this field, existing methods still suffer from two fundamental limitations in characterizing path strength:

First, the neglect of the attenuation effect of intermediary node quantity on propagation strength. Current “best path” methods assume that path strength is determined solely by the weakest connection in the path (bottleneck principle), without considering the impact of intermediary node quantity itself on the path strength. As illustrated in Fig 3, the direct path a (1 → 4) and the path b (1 → 2 → 4) passing through one intermediary node from Actor1 to Actor4 are assigned identical fuzzy influence strengths under existing methods [15]. However, extensive empirical evidence [33,35,44,45] demonstrates that information or social influence in real-world networks exhibits a decreasing trend as the number of intermediary nodes increases. Neglecting this fundamental principle may lead to systematic overestimation of influence strength for long-distance, multi-hop paths, thereby affecting the accuracy of network analysis.

Second, the neglect of the moderating role of intermediary node characteristics on propagation intensity. Existing methods conceptualize influence propagation as a purely “pipeline” process, assuming intermediary nodes function merely as passive transmission channels [15]. However, social network theory demonstrates that intermediary node characteristics exert substantive influence on propagation processes [44,46]. In the critical issue of path strength characterization, this moderating effect manifests primarily through differential mechanisms of tie strength. As illustrated in Fig 3, paths b (1 → 2 → 4) and c (1 → 3 → 4) from Actor1 to Actor4 exemplify the specific operation of this mechanism. The differential propagation effects between these paths stem not merely from numerical comparisons of connection strengths TD/TC, but more fundamentally from the qualitative characteristics of Actor1’s relationships with each intermediary node. When a strong tie exists between Actor1 and Actor2, this relationship enhances influence propagation through multiple mechanisms: ① trust-based deep interaction facilitates the preservation of information integrity; ②frequent communicative contact creates diversified influence channels; ③shared cognitive frameworks reduce information distortion during propagation. Conversely, the weak tie between Actor1 and Actor3, lacking these reinforcement mechanisms, may significantly constrain effective influence transmission. This observation resonates with classical research in social network theory. While Granovetter’s (1973) [9] “strength of weak ties” theory primarily emphasizes the unique value of weak ties in information diffusion, it simultaneously acknowledges the crucial role of strong ties in deep influence and complex information transmission. Hanneman [44], through systematic network analysis methodology, confirms that actors connected through shorter path distances typically maintain stronger actual connection intensities, providing a structural explanation for understanding the moderating function of intermediary nodes. Jackson [33–34] reveals these nonlinear decay characteristics in his Decay Centrality research, establishing a theoretical foundation for quantifying the moderating effects of intermediary nodes. Therefore, ignoring the strength adjustment role of intermediary nodes is not only a methodological limitation, but also affects the accurate grasp of the structural characteristics of fuzzy networks.

These limitations highlight the gap between existing methods and actual social network behaviors, underscoring the urgent need to develop a more comprehensive theoretical framework that accurately captures the complex characteristics of path strength in fuzzy networks, thereby establishing a solid foundation for subsequent calculations of betweenness centrality and closeness centrality.

(2) **Redefining the “ Fuzzy Intensity of Path”**

Based on the above considerations, we will redefine the description framework for the “best path.” First, we will redefine the **Fuzzy Intensity of Path**:

**Definition 4.2. Fuzzy intensity of path (FIP)**.Suppose $v_{i}\cdot {\tilde{A}}_{ih1}\cdot v_{h1}\cdot {\tilde{A}}_{h1h2}\cdot ...\cdot v_{h_{s}}\cdot {\tilde{A}}_{h_{s}j}\cdot v_{j}$ represents a valid path from *i* to *j*, where *h* denotes intermediary nodes in the transmission process. To characterize the complex features of path strength, we decompose the influencing factors into two key functions:①Intermediary strength function r(·) quantifies the extent of influence during indirect transmission;②Intermediary node decay function ρ(·) models the decay of influence as the number of intermediary nodes increases.The fuzzy intensity of this path is defined as $FIP_{ij}^{i\to h\to j}$:


$$FIP_{ij}^{i\to h_{1}\to ...\cdot h_{s}\to j}=\tilde{f}({\tilde{B}}_{l,l+1},r(\cdot ),\rho (\cdot )|l\in \{i,h_{1},h_{2},...,h_{s},j\},h\neq \{i,j\})$$ (22)

where ${\tilde{B}}_{l,l+1}$ is the value of ${\tilde{A}}_{l,l+1}$ after normalization using Equation (23).

Formula (22) provides a general framework for characterizing the strength of fuzzy paths. To ensure theoretical soundness and practical operability of the model, this study constructs the path strength framework based on three core assumptions:

① Bottleneck Effect: The overall strength of a path is limited by its weakest connection. This principle has been widely recognized in fuzzy network analysis literature [15], ensuring that path strength does not exceed its weakest link.
② Indirect Propagation Upper Bound: The influence strength of indirect connections should not exceed that of direct connections. This conforms to the fundamental laws of information propagation, ensuring logical consistency of the model and preventing unreasonable amplification of indirect influence.
③ Distance Decay Effect: Influence decays as the number of intermediary nodes increases. This phenomenon has been fully validated in empirical studies of social networks [33–35,45], capturing the natural decay characteristics of information propagation in real networks.

Based on these assumptions, we concretize the general framework into the following computational model. It should be clarified that there is no unique solution for modeling fuzzy path intensity—different concretization schemes may be suitable for different network characteristics and application scenarios. The computational model proposed in this study represents an attempt to balance theoretical rigor with practical operability. The value of this model lies in: (1) providing a systematic framework to integrate the effects of intermediary node quantity and node characteristics on path strength; (2) enabling the model to adapt to different types of fuzzy social networks through adjustable parameters; (3) more accurately reflecting the complex characteristics of path strength compared to existing methods while maintaining computational efficiency. Subsequent theoretical analysis and empirical cases will demonstrate how this framework effectively captures path strength characteristics in fuzzy social networks and provides researchers with a flexible and extensible analytical tool.


$$FIP_{ij}^{\prime }{}^{i\to h_{1}\to ...\cdot h_{s}\to j}=\{\begin{array}{c} {}*20l{\tilde{B}}_{ij};\;\;\;\;\;\;\;\;\;\;\;\;h_{s}=0\\ \min_{GOC}\left\{\frac{{\left({\tilde{B}}_{l,l+1}\right)}^{r(f_{1}(l,l+1),\beta_{1}(Gf))}}{{\left(h_{s}+1\right)}^{\rho (f_{2}(i,h_{1},h_{2},...,h_{s},j),\beta_{2}(Gf))}}|l\in \{i,h_{1},h_{2},...,h_{s},j\}\right\};\;\;\;\;\;\;h_{s}\neq 0 \end{array}$$ (23)

In the formula, FIP are divided into two cases: without intermediary nodes and with intermediary nodes

① **Without Intermediary Nodes**

In this case, the FIP between nodes is directly equal to the assignment fuzzy relation of two nodes. This arises because, when focusing on the pairwise direct relationships between nodes, the Typical connection framework adequately accounts for the influence of node heterogeneity and the inherent fuzziness of relationships.

② **With Intermediary Nodes**

To satisfy the bottleneck effect, path strength is determined by the edge with minimum strength. The function $r(f_{1}(l,l+1),\beta_{1}(G_{f}))$ represents the intermediary strength function, incorporating two components: ***i)*** Relationship characteristics between nodes *l* and *l + 1*, genera*l*ized as $f_{1}(l,l+1)$; ***ii)*** Impact of network structure on indirect influence propagation capability, generalized as $\beta_{1}(G_{f})$. Similarly, the function $\rho (f_{2}(i,h_{1},h_{2},...,h_{s},j),\beta_{2}(G_{f}))$ corresponds to the intermediary node decay function, further decomposed into: ***i)*** Path topology characteristics, generalized as $f_{2}(i,h_{1},h_{2},...,h_{s},j)$; ***ii)*** Network-induced attenuation effects from intermediary node quantity, generalized as $\beta_{2}(G_{f})$.

Equation (23) provides a general theoretical framework for characterizing fuzzy path intensity (FIP). However, directly applying this framework faces significant challenges in practice: on one hand, precisely defining the intermediary strength function and node decay function for each path requires extensive domain knowledge and empirical data; on the other hand, as network scale expands, the computational complexity of individual modeling becomes prohibitive. To balance the theoretical completeness and practical operability of the model, this study adopts a parameterized simplification strategy. The core of this strategy lies in abstracting complex node heterogeneity and network structure effects into global parameters, thereby avoiding individualized modeling for each node. While this approach reduces model granularity to some extent, it significantly enhances its practicality and scalability. Based on this approach, we simplify the general framework into the following form:


$$FIP_{ij}^{\prime \prime }{}^{i\to h_{1}\to ...\cdot h_{s}\to j}=\{\begin{array}{c} {}*20l{\tilde{B}}_{ij};\;\;\;\;\;\;\;\;\;\;\;\;h_{s}=0\\ \min_{GOC}\left\{\frac{{\left({\tilde{B}}_{l,l+1}\right)}^{r}}{{\left(h_{s}+1\right)}^{\rho }}|l\in \{i,h_{1},h_{2},...,h_{s},j\}\right\};\;\;\;\;\;\;h_{s}\neq 0 \end{array}$$ (24)

In the formula, *r* and ρ are simplified as constants determined by the global network structure, with values dependent on node types and relationship types. Here, *r (r ≥ 1)* quantifies the blocking strength of intermediary nodes in influence propagation. A higher *r* indicates that intermediary nodes are less capable of efficiently propagating influence to other nodes. Specifically, when *r* = 1, no attenuation occurs during influence propagation due to the inherent properties of intermediary nodes. Notably, the case of *r* < 1 would imply that indirect paths could be stronger than direct connections, which violates fundamental principles of information propagation and is therefore excluded from the model. The parameter ρ(ρ≥0) measures the impact of the number of intermediary nodes on propagation efficiency. A larger ρ implies a stronger inhibitory effect of increasing intermediary nodes on efficiency, reflecting the limited ability of nodes to propagate influence across multiple intermediaries. Notably, when ρ=0, the influence remains unaffected by the number of intermediary nodes in the path.

Compared to existing research methods, this model offers two significant advantages: (1) it more accurately captures realistic constraints of path strength in fuzzy networks while maintaining computational efficiency; (2) it provides flexible modeling capability through adjustable parameters, enabling researchers to make adaptive adjustments based on specific network characteristics and application requirements, better meeting practical operational needs.To illustrate with a simple example in Example.1

**Example 1. Demonstration Example of FIP**

**Fig 4:**
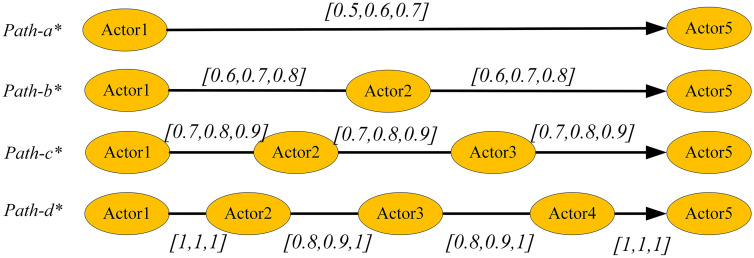
Four paths from actor1 to actor 5.

**Fig 5:**
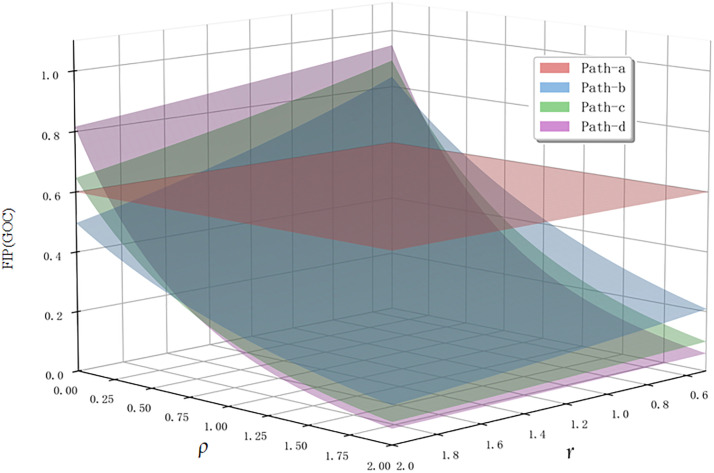
Variation of path intensity centroid values for example paths under different parameter conditions.

As shown in Fig 4, consider four paths from Actor1 to Actor5, where the relative strengths of these paths are not immediately apparent and cannot be easily determined through intuitive observation. Table 2 provides the numerical values of fuzzy path intensity calculated using the centroid method under different parameter settings, while Fig 5 visualizes the dynamic characteristics of path strength variations with parameters through three-dimensional surfaces.

**Table 2:** FIP when different values of *r* and ρ are used.

Case/GOC	C1	C2	C3	C4	C5	C6	C7	C8
	*r* = 1 *ρ* = 0	*r* = 1.5 *ρ* = 0	*r* = 2 *ρ* = 0	*r* = 1 *ρ* = 0.5	*r* = 1 *ρ* = 1	*r *= 1 *ρ* = 1.5	*r* = 1.5 *ρ* = 1	*r* = 2 *ρ* = 1.5
*FIP*_*(Path-a)*_	0.6	0.6	0.6	0.6	0.6	0.6	0.6	0.6
*FIP*_*(Path-b)*_	0.7	0.59	0.5	0.5	0.35	0.247	0.294	0.176
*FIP*_*(Path-c)*_	0.8	0.71	0.65	0.462	0.267	0.154	0.239	0.124
*FIP*_*(Path-d)*_	0.9	0.86	0.82	0.45	0.225	0.113	0.214	0.102

By adjusting parameters *r* and *ρ*, we can observe different variation patterns in path strength, reflecting the diverse characteristics of influence propagation under different network environments:

(1) Parameter Sensitivity Analysis: As *r* and *ρ* values change, the relative strength relationships among the four paths undergo significant alterations. This dynamic variation reveals an important fact—when considering node heterogeneity and propagation decay effects, path strength is no longer static but depends on our understanding and modeling of network characteristics. This provides researchers with the flexibility to adjust the model according to specific network features.
(2) Comparison with Existing Methods: Particularly noteworthy is that when *r* = 1 and *ρ* = 0 (Case C1 in Table 2), the model degenerates to the simplified form widely adopted in existing literature, which does not consider the decay effects and strength loss of intermediary nodes. The inclusion of this special case demonstrates that our framework is a natural extension of existing methods rather than a replacement.

By introducing the parameterized concept of fuzzy path intensity, this study provides richer modeling capabilities while maintaining compatibility with existing methods, enabling researchers to more accurately characterize the complex features of influence propagation in real-world social networks.

(2) **Best path.**

**Definition 4.3**. Best path. Suppose there are *k* paths between node *i* and node *j*, then fuzzy intensity of the best path can be expressed as:


$$BFIP_{ij}=\max{\mathrm{\backslash nolimits}}_{GOC}\left\{FIP_{ij}^{\left(1\right)},FIP_{ij}^{\left(2\right)},...,FIP_{ij}^{\left(k\right)}\right\}$$ (25)

Where, $\max_{GOC}$ represents the process of ranking fuzzy numbers using the centroid method and selecting the maximum value among the fuzzy numbers as the best path between node *i* and node *j*. This method effectively reflects the strongest influence relationship between nodes, making the quantification of path strength in the network more realistic.

(3) **Fuzzy Betweenness Centrality**

$${\tilde{C}}_{B}\left(v_{i}\right)=\sum_{s\neq t\neq v_{i}}\frac{\sigma_{st}\left(v_{i}\right)}{\sigma_{st}}$$ (26)

Where ${\tilde{C}}_{B}\left(v_{i}\right)$ denotes the betweenness centrality of node *v*_*i*_$\sigma_{st}$ represents the total number of best paths between node *s* and node *t*$\sigma_{st}\left(v_{i}\right)$ represents the number of best paths between node *s* and node *t* tha*t* pass through node *v*_*i*_.

#### 4.2.3. Fuzzy closeness centrality.

(1) **The limitations of the existing fuzzy closeness centrality**

Closeness centrality measures the proximity of a node to other nodes in a network, serving as a crucial metric for assessing a node’s overall reachability. In classical SNA, closeness centrality is typically calculated based on shortest paths. For fuzzy networks, existing research primarily adopts the “best path” approach [15], which selects the single path with maximum influence strength to measure proximity between nodes. This method performs well in scenarios with clear path selection.

However, relying solely on the best path method presents fundamental limitations when characterizing genuine proximity relationships in fuzzy social networks. In real-world social networks, influence between nodes often propagates simultaneously through multiple paths, exhibiting significant cumulative effects. As Hanneman [44] observes: “Multiple connections may indicate that the tie between two actors is stronger than a single connection.” This observation reveals the multidimensional nature of network relationships—actual proximity between nodes results from the combined effects of multiple paths rather than being determined solely by the strongest path.

Taking Fig 3 as an example, suppose Actor1 connects to Actor4 only through path a, with influence expressed as *influence*(1). When Actor2 joins the network, Actor1 can influence Actor4 not only through path a but also through path b. While existing methods [15] typically ignore path b due to its lower strength compared to path a, in real social network environments, the combined effect of both paths, *influence*(2), usually exceeds *influence*(1). This phenomenon has been validated in dynamic diffusion model studies [46–47]: influence between nodes often manifests as collective interaction processes, where the synergistic effects of multiple paths more effectively capture overall influence strength between nodes, highlighting the importance of “community effects” in social networks.

It should be clarified that identifying limitations of the single best path method does not negate its value. Indeed, the research by Porreca et al. [15] and Hu et al [11] holds significant importance: (1) the best path has strong representativeness in certain specific scenarios; (2) it can intuitively reveal strong correlations in networks; (3) it serves as an important benchmark for assessing node proximity. However, to comprehensively describe proximity relationships between nodes in fuzzy networks, it is necessary to systematically consider the cumulative contributions of multiple paths while acknowledging the special status of the best path.

(2) **Introduction of the Concept of TFIP (Total Fuzzy Intensity of Path)**

To this end, in order to accurately assess the true closeness relationship between nodes based on the aggregation of multiple paths, this study introduces the concept of TFIP in the calculation of closeness centrality.

**Definition 4.4**. **Total Fuzzy Intensity of Path (TFIP)**. Suppose there are *k* paths between node *i* and node *j*$TFIP_{ij}$ represents the combined effect of all path influences between the two nodes, rather than selecting only the single Best Path. The specific formula is as follows:


$$TFIP_{ij}=\{(FIP_{ij(1)},FIP_{ij(2)},...,FIP_{ij(k)}),U_{ij}(\cdot )\}$$ (27)

Here, $U_{ij}(\cdot )$ represents an aggregation operator that satisfies the requirements of $TFIP_{ij}$, facilitating the aggregation of fuzzy path influences. It is worth noting that selecting an appropriate aggregation method is itself an open research question, as different aggregation strategies may be suitable for different network characteristics and application scenarios. In exploring effective path aggregation mechanisms, this study proposes the following working assumptions based on empirical observations of network behavior and theoretical guidance from relevant literature [11,15,44,46]:

① Path Independence: The strength calculation of each path is mutually independent without interference effects. This assumption simplifies complex path interactions into a superposition of independent contributions. While this simplification may overlook synergistic or competitive effects between paths, it enables the model to capture fundamental characteristics of multi-path propagation while maintaining computational feasibility.
② Ranking Importance: After paths are sorted in descending order by strength, their ranking positions affect their contributions to overall proximity. This assumption is grounded in priority principles from decision theory, reflecting cognitive patterns in processing multiple information sources. This also provides the theoretical foundation for why literature [11,15] considers only the optimal path.
③ Marginal Diminishing Effect: As the number of considered paths increases, the contribution of additional paths to the overall influence exhibits a diminishing trend. Drawing from the principle of diminishing marginal utility in economics, this assumption reflects the limited nature of attention resources and the selective characteristics of information processing in real-world networks.

Based on these assumptions, we construct the following parameterized aggregation framework:


$$U_{ij}^{\prime }=g(\theta (\cdot ),z(\cdot ))$$ (28)

where *θ(·)* is a k-dimensional weight vector reflecting the relative importance of different paths. In practical applications, the best path typically carries the primary influence, yet the cumulative effects of secondary paths cannot be overlooked. *z(·)* is a decay vector that characterizes the pattern of path influence decline with ranking position. This decay may exhibit linear, exponential, or other patterns, with the specific form depending on network characteristics.

To transform the conceptual framework into an operational computational model, this study proposes a parameterized implementation scheme. It should be clarified that this scheme is not a unique solution, but rather one specific implementation based on practical feasibility considerations:


$$U_{ij}^{\prime \prime }=\left\{\theta_{1},\theta_{2}\times {\left(\frac{\text{1}}{\text{2}}\right)}^{z},...,\theta_{2}\times {\left(\frac{\text{1}}{k}\right)}^{z}\right\}$$ (29)

Correspondingly, the total path intensity can be expressed as:


$$TFIP_{ij}=\underset{t=1}{\overset{k}{⊕}}U_{ij}^{\prime \prime }⊙FIP_{ij(t)}^{e}$$ (30)

where, $FIP_{ij(t)}^{e}$ represents the fuzzy intensity of the t-th path from node i to node j, sorted in descending order by strength; the fuzzy number aggregation method is detailed in Section 3.3.2.

This parameterized design exhibits the following characteristics: ① $\theta_{1}$ and $\theta_{2}$ control the influence weights of the best path and secondary paths respectively, facilitating understanding and adjustment. ②Flexibility: By adjusting parameter values, one can select appropriate settings within a continuous spectrum from “considering only the best path” (θ₂ = 0) to “all paths equally important” (θ₁ = θ₂, z = 0).

Regarding the setting of the decay coefficient z, this study provides a power-law based scheme: ${\left(\frac{\text{1}}{k}\right)}^{z}$. This choice is inspired by the ubiquity of power-law distributions in network science, while also providing rich modeling possibilities for different scenarios:

① **when *z* > 1:** $\sum_{i=1}^{k}{\left(\frac{\text{1}}{k}\right)}^{z}$ converges and has an upper limit, it indicates that even with an infinite number of low-intensity secondary paths being accumulated, the influence relationship does not grow without limit. The larger *z*, the smaller the total amplification, the more reflecting scenarios where secondary paths are less important.
② **When *z* = 1**: The decay coefficient for secondary paths follows a harmonic series $\sum_{i=1}^{k}\frac{\text{1}}{k}$, which is divergent. This case indicates that the secondary path length continuously increases, with no upper limit for the influence relationship. However, as the number of paths increases, the impact increases more slowly. At this point, it reflects that while the secondary path can increase the proximity between two nodes without limit, its marginal effect decreases significantly.
③ **When *z* < 1:** $\sum_{i=1}^{k}{\left(\frac{\text{1}}{k}\right)}^{z}$ diverges faster, and the importance of secondary paths is magnified. At this point, even paths ranked lower can still play a significant role in determining node proximity, which is applicable to scenarios that highlight the significance of secondary paths.
④ **When *z* = 0**: All paths are equally weighted in secondary paths. In this scenario, if $\theta_{1}$ = $\theta_{2}$, it indicates that all paths are considered equally important in the *TFIP* calculation.

**Fig 6:**
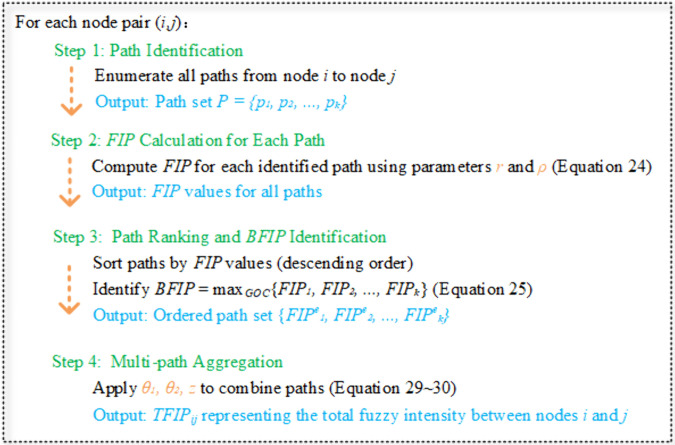
TFIP computation process.

Based on the above discussion, Fig 6 presents the computational workflow for determining TFIP values between node pairs.

Based on *TFIP*, it can realistically reflect the mutual influence relationships between nodes across the entire network.

It should be emphasized again that the *TFIP* framework proposed in this study represents an exploratory attempt aimed at providing a more flexible and comprehensive analytical tool for FSNA. The value of this method lies not in providing a uniquely correct solution, but rather in its supplementation and extension of existing methodological systems: ①Expanding analytical perspectives: Extending from single best path to multi-path aggregation, more comprehensively characterizing the complex relationships between nodes, helping to reveal network structural features that traditional methods might overlook.②Providing an adjustable framework: Through parameterized design, enabling researchers to flexibly customize analytical methods based on the structural characteristics of specific networks, domain expertise in application fields, and specific requirements of research objectives.③Stimulating methodological innovation: Providing theoretical foundations and empirical references for exploring more refined aggregation mechanisms, more reasonable parameter selection strategies, and more domain-specific specialized methods.

(3) **Fuzzy Closeness centrality**

Based on the *TFIP*, the closeness centrality in a fuzzy network can be expressed as follows:


$${\tilde{C}}_{c}^{in}\left(v_{i}\right)=\underset{j\in N,j\neq i}{⊕}\left(q_{j}^{a}⊙TFIP_{ji}\right)$$ (31)

$${\tilde{C}}_{c}^{out}\left(v_{i}\right)=\underset{j\in N,j\neq i}{⊕}\left(q_{j}^{a}⊙TFIP_{ij}\right)$$ (32)

$${\tilde{C}}_{c}^{tot}\left(v_{i}\right)={\tilde{C}}_{c}^{in}\left(v_{i}\right)+{\tilde{C}}_{c}^{out}\left(v_{i}\right)$$ (33)

where ${\tilde{C}}_{c}^{in}\left(v_{i}\right)$ represents fuzzy in-Closeness Centrality; ${\tilde{C}}_{c}^{out}\left(v_{i}\right)$ represents fuzzy out-Closeness Centrality; ${\tilde{C}}_{c}^{tot}\left(v_{i}\right)$ represents fuzzy total-Closeness Centrality; To unify the scale and facilitate comparisons across different networks, it is suggested that $q_{j}^{a}$ be set as $\frac{\text{1}}{n-1}$.

**Example 2. Demonstration Example of Fuzzy Closeness centrality with TFIP.**

**Fig 7:**
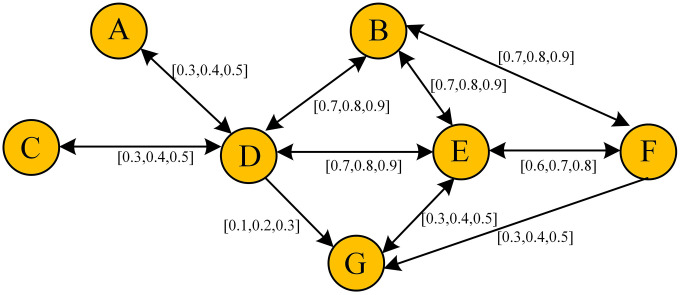
A fuzzy interpersonal relationship network in example 2.

To illustrate the application potential of the fuzzy closeness centrality method proposed in this paper, a small-scale directed fuzzy network with 7 nodes is presented as an example. This network exemplifies a typical interpersonal relationship structure, constructed through modeling the cooperative interactions within a specific department of a school. The relationships of all edges in the network are represented using TFN. See Fig 7 for details.

First of all, it is necessary to assess its closeness centrality. Based on TFIP, we have configured nine distinct parameter sets to simulate various scenarios, with details presented in the Table 3.

**Table 3:** Ranking of node closeness centrality under different.

Scenario		Ranking of Node Closeness Centrality
S1: Directed Unweighted Network.		D > E > B > G > F > C = A
S2 Without Considering the Decay of Fuzzy Intensity Between Nodes(r = 1,p = 0).	S2.1 All Paths Are Equally Important. ($\theta_{1}=1,\theta_{2}=1,z=0$)	F > B > E > G > D > C = A
	S2.2 Decreasing Weights of Secondary Paths. ($\theta_{1}=1,\theta_{2}=1,z=1$)	F > B > E > D > G > C = A
	S2.3 Reduced Importance and Decreasing Weights of Secondary Paths. ($\theta_{1}=1,\theta_{2}=0.5,z=1$).	F > B > E > D > G > C = A
	S2.4 Considering Only Best Paths. ($\theta_{1}=1,\theta_{2}=0$)	B = D > E = F > C = A = G
S3 Considering the Decay of Fuzzy Intensity Between Nodes(r = 1.2,p = 1).	S3.1 All Paths Are Equally Important. ($\theta_{1}=1,\theta_{2}=1,z=0$)	B > E > F > D > G > C = A
	S3.2 Decreasing Weights of Secondary Paths. ($\theta_{1}=1,\theta_{2}=1,z=1$)	E > B > D > F > G > C = A
	S3.3 Reduced Importance and Decreasing Weights of Secondary Paths. ($\theta_{1}=1,\theta_{2}=0.5,z=1$).	E > B > D > F > G > C = A
	S2.4 Considering Only Best Paths. ($\theta_{1}=1,\theta_{2}=0$)	E > D > B > F > G > C = A

Scenario 1 employs a directed unweighted network structure. Scenarios 2.1 to 2.4 are based on the assumption that path intensity is independent of intermediate nodes, and are further categorized as follows: S2.1 assumes all paths are equally important; S2.2 considers the Best Path as the primary factor in closeness relationships, with secondary paths retaining some importance; S2.3 prioritizes the Best Path for closeness relationships, while the importance of secondary paths is diminished; and S2.4 exclusively considers the Best Path as the primary factor in closeness relationships. Scenarios 3.1 to 3.4 are predicated on the assumption that the path intensity of indirect transmission is influenced by intermediate nodes, with all other conditions aligned with those of Scenarios 2.1 to 2.4.

Based on the data analysis from Table 3, the ranking of network closeness centrality exhibits certain variations under different parameter conditions. In Scenario 1, which employs a simplified model and does not consider the impact of connection strength, Node D has the highest importance, followed by Node E. Scenario 2.4 corresponds to the settings adopted by Porreca et al [15] and Hu et al [11], representing a special case within the FTIP framework. In this scenario, node rankings lack effective differentiation, with nodes B and D sharing identical closeness centrality values, and nodes E and F exhibiting equivalent closeness centrality. Notably, this scenario fails to adequately address the dynamic variations in path intensity and the potential influence of secondary paths.

In other experimental scenarios, adjustments to parameters lead to observable differences in closeness centrality. This phenomenon aligns with the understanding of real-world networks, where node centrality is not fixed but varies when accounting for actual differences in node types and the diversity of network relationships. Scenario 2.3 is particularly noteworthy due to its strong representativeness. Its parameter settings were derived from a consensus among domain experts and evaluators regarding core concepts, validated through simplified scenarios involving 2–3 nodes, and the final resulting closeness centrality ranking was affirmed by participants. In Scenario 3.3, nodes E, B, and D rank highest in closeness centrality, showing some consistency with the findings of Scenario 2.4. However, the significant elevation in node E’s ranking indicates that its importance in assessing closeness relationships deserves greater attention. Nevertheless, this study reveals that current parameter selection predominantly relies on expert consensus regarding network properties, requiring validation through predefined parameters and node-based verification. This underscores the need for future research to establish a standardized parameter framework tailored to diverse application scenarios.

**Fig 8:**
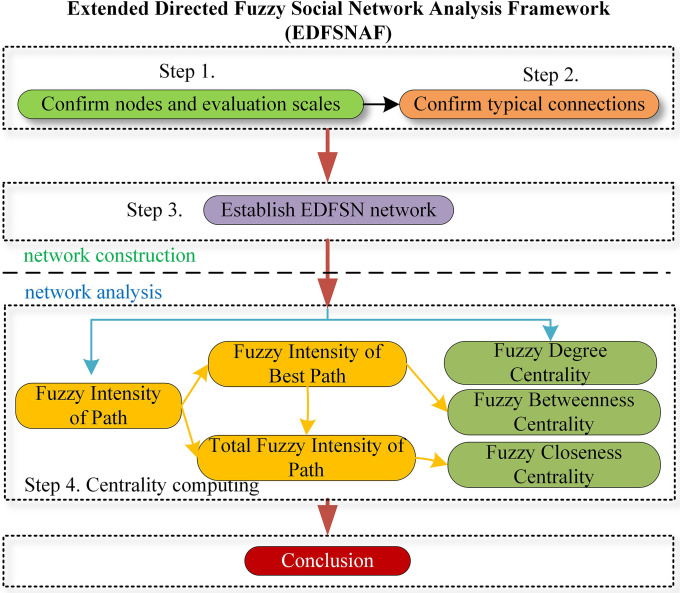
Flow chart of EDFSNAF.

**The application process of the EDFSNAF is shown in Fig 8**.

In some typical fields of SNA, such as collaboration networks, diffusion of information, social networks of books sold by online bookstores [48], and accident analysis [6] —— there exists a consistent and pressing need to construct targeted networks and identify core nodes. FSNA offers a deeper level of authenticity and precision in modeling complex systems influenced by human factors. As precision improves, the characteristics of relational ambiguity and network heterogeneity become increasingly prominent. To address this, the EDFSNAF proposed in this section represents a proactive attempt to construct fuzzy social networks and pinpoint critical nodes under conditions of uncertainty and diverse network types. This section validates the potential of this method using a simple personnel collaboration network as an example. In the subsequent case study section, we will expand the scope of our research. Given the recent profound transformations in Chinese vocational education—where nationally standardized professional curricula have become mandatory guidelines for relevant disciplines—this significant shift warrants further exploration of its underlying potential and challenges. The EDFSNAF framework introduced herein serves as a suitable tool for this analysis. On another note, compared to conventional interpersonal networks, this network not only integrates typical elements of personnel participation evaluation but also exhibits significant node heterogeneity, making it an ideal testbed for demonstrating the applicability and effectiveness of EDFSNAF in Fuzzy SNA.

### 4.3. Ethical considerations

To ensure the integrity and ethical soundness of this empirical investigation, appropriate ethical considerations were implemented. The School of Rail Transit at Yunnan Transportation Vocational College confirms that, due to complete data anonymization and its classification as a routine teaching activity, this study does not require formal ethical review. All participants voluntarily joined the study between January 15, 2025, and February 15, 2025, and signed written informed consent forms. Participants did not receive any form of compensation, incentives, or rewards for their involvement in this study, ensuring that their participation was entirely voluntary and free from any external influence. The consent forms clearly outline the study’s purpose, procedures, and participants’ rights, and are securely archived by the research team. Data were anonymized prior to analysis, and the study fully complies with international ethical standards. These measures ensure that the empirical validation maintains both scientific rigor and ethical responsibility while demonstrating the practical applicability of the EDFSNAF framework in real-world educational contexts.

## 5. Case study

### 5.1. Case background and network construction basis

(1) **Rationality Analysis of China’s Vocational Education Curriculum Network Construction**

Curriculum is a framework that expresses goals, expectations, and means of realization, and it is an important tool for schools to guide students toward comprehensive development [49]. Generally, Course design is characterized by flexibility and variability. However, with the rapid development of vocational education in China, to address disorderly expansion and promote standardized construction, the State Council and the Ministry of Education have successively issued a series of national teaching standards for vocational education. These standards explicitly specify the requirements for the major name and the design of core courses [50–53]. Based on this framework, different regions and vocational colleges add course content tailored to their own circumstances, ultimately forming unique curriculum systems for each major.

By enforcing major standards, it is possible to achieve standardized and unified curriculum design, thereby preventing vocational colleges from blindly adding new majors or excessively expanding enrollment due to profit-driven motives without sufficient conditions. However, major standards are not static; they are periodically revised at the national level based on actual needs. At the same time, Vocational colleges continuously adjust their curricula at different stages to align with national major standards, market conditions, and their own circumstances. During the long-term dynamic adjustment of the curriculum, discrepancies in how experts at different levels understand the majors may arise when designing the curriculum, potentially leading to the following issues:

**(1) Lack of correlation between courses**: course content lacks logical connections, making it difficult to form a systematic knowledge structure; **(2) Repetition of knowledge content:** Significant overlaps exist between different courses, wasting educational resources; **(3) Inability to identify key courses from a holistic perspective:** The failure to identify and define key courses within the curriculum system from a systemic perspective disrupts the logical structure of teaching; **(4) Uneven resource allocation:** Key courses fail to receive priority resource allocation, negatively impacting teaching outcomes.

Therefore, the **EDFSNAF** developed in this study holds significant practical value for optimizing the vocational education curriculum system in China. This method can systematically analyze the structure of curriculum systems, identify key courses, and optimize resource allocation, thereby promoting the standardization and scientific construction of vocational education curricula.

(2) **Characteristics of Curriculum Networks**

The curriculum system can be mapped as a curriculum network, where courses are represented as nodes of the network and the relationships between courses as edges. Since the connections between courses are influenced by multiple uncertain factors [49,54–56] these connections typically exhibit fuzziness, primarily determined by subject characteristics, knowledge associations, and other factors.

**Fig 9:**
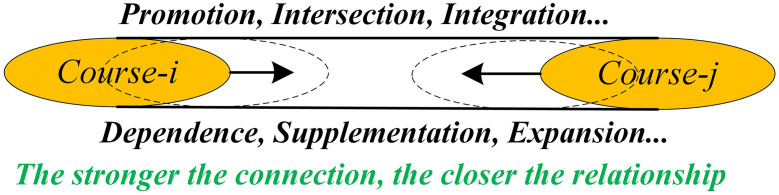
The relationship between courses.

As the relationships between courses become closer, their interactions also strengthen. However, the interaction between courses is not always symmetric. For example, the influence of course *i* on course *j* may differ from the influence of course *j* on course *i*. This asymmetry makes traditional undirected networks insufficient to fully describe the interrelationships within the curriculum system. Therefore, the course network can be modeled as a fuzzy directed network to better represent the complex relational structure within the curriculum system. The relationships between courses are detailed in Fig 9.

(3) Case Selection

The case studies were selected from the curriculum system of urban rail transit-related majors at a vocational college in China. As one of the typical areas of Chinese vocational education, by 2024, more than 230 colleges in China have established urban rail transit-related majors. For this study, the **Urban Rail Transit Operation and Management Major** (URTOMM) and the **Urban Rail Transit Communication and Signaling Technology Major** (URTCSTM) from the same college were selected. The following text primarily presents the process of constructing and analyzing the curriculum network for the URTOMM, while the URTCSTM is presented as a comparison in the comparative analysis section.

Both majors are foundational specialties in the field of urban rail transit: URTOMM focuses on on-site management work during the operation of urban rail transit, aiming to cultivate applied talents with management and organizational capabilities. URTCSTM focuses on the maintenance of communication and information systems, aiming to develop professionally skilled talents proficient in rail communication technologies [52].

By analyzing the curriculum networks of these two majors, the study aims to identify the core courses and their positions within the overall curriculum system, while also uncovering the structural relationships between courses. This analysis will provide a scientific basis for curriculum optimization and resource allocation. For the evaluation, 4 professional instructors and 2 employees from urban rail transit enterprises were selected to participate in the study to ensure the scientific validity and practical relevance of the results.

### 5.2. The curriculum network construction

#### 5.2.1. Construct typical connections and parameter selection.

Based on expert consensus, the intensity of the connections between courses is represented by a score ranging from *0* to *3*, followed by the establishment of typical connections.

(1) **Construct typical connections**

**Step.1** Formation of the preliminary set of typical connections.

**Step.1.1** Define noninteractive connection: Based on the concept of maximum distance, a type of connection is defined, which is typically categorized as no connections.

**Step.1.2** Identify the closest course connection: As the major’s background, application equipment, and theoretical foundation of course nodes become increasingly aligned, their connections grow tighter. Since identical courses are rare, the closest connections are defined as “highly close connection.”

**Fig 10:**
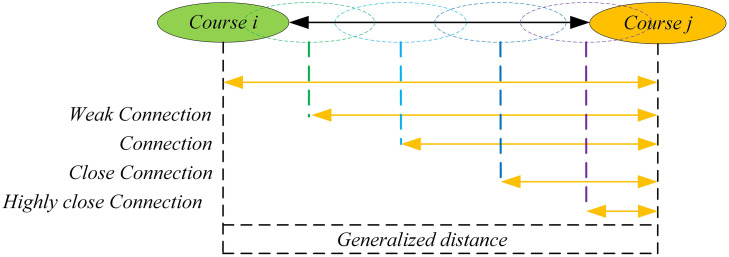
Analysis of the degree of closeness in the links among courses.

**Step.1.3** Fuzzy classification of connections: In the curriculum network, connections are inherently fuzzy, lying between no connection and highly close connection. Based on the degree of interaction and typicality of courses, three types of fuzzy connections are defined: weak connection, general connection, and tight connection. A detailed illustration is shown in Fig 10.

**Step.2 Detailed Description and Refinement** Characterize different connection types precisely, and clarify the basic forms of their effects. When connections of the same degree exhibit different forms of interaction, further refinement in classification is required. Subsequently, representative examples of different typical connections are presented to better illustrate the practical applications and characteristics of each connection type. The detailed information of typical connections in the curriculum network is shown in **Table 4**.

**Table 4:** Table of typical connections in curriculum networks.

Typical connection	Explanation of the connection	Illustrations of typical connections (from *i* to *j*)
TYPE-A No Connetion	It focuses on the development of students’ abilities in various aspects and levels, but does not establish a clear connection in terms of subject categories or specific directions of skill development.	“Current Situation and Policy” –”Train Operation Organization of Urban Rail Transit(URT)”
TYPE-B Weak Connection	Although there is a certain degree of overlap between the subjects, there is no clear relationship between the knowledge areas, and no substantial interaction in the direction of specific ability development.	“ Professional Ethics of URT”~ “Train Operation Organization of URT “
TYPE-C Connecion	There is a limited amount of overlap in certain knowledge points, or minor sequential relationships between courses. The primary characteristic is that they work together from various perspectives to foster the development of a specific skill.	“Fundamentals of Computer Application” - “URT Communication and Signaling” AND “Urban Rail Transit Lines and Stations”-“Passenger Organization of URT”
TYPE-D Close connection	As the connections deepen, connections of the same degree exhibit different typical characteristics. In this regard, the close relationships can be further divided into: D1, D2, and D3.	
TYPE-D1	The courses present knowledge overlap on a chapter basis, but the degree of repetition is low, with each course having its own focus.	“Railway Special Communication Equipment” - “Railway Mobile Communication System”
TYPE-D2	Course *j* is briefly outlined within course *i*, but course *j* offers a more systematic and comprehensive approach. This connection is mainly reflected in the relationship between introductory courses and core skill courses.	“Passenger Service of Urban Rail Transit”-”Automatic Fare Collection System and Ticket Handling of Urban Rail Transit”
TYPE-D3	The teaching content of Course *i* and Course *j* has a clear and strong sequential order.	“Train Operation Organization of Urban Rail Transit”-”Comprehensive Training on Station Affairs”
TYPE-E Highly Close Connetion	The teaching content of Course *i* and Course *j* shows a high degree of overlap, with consistent knowledge structures and significant substitutive effects.	“English for URT Passenger Service”-” Specialized English for URT”

**Fig 11:**
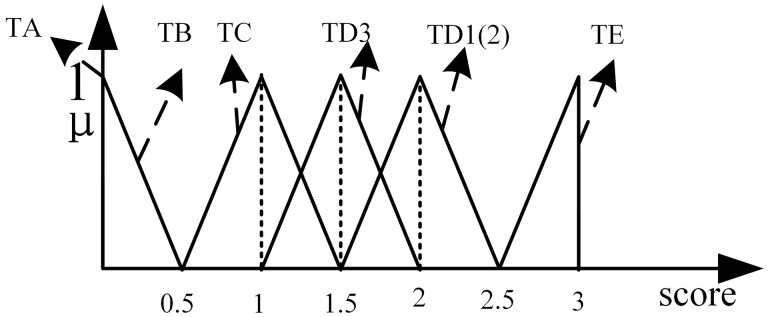
Fuzzy set of typical connections.

Step.3 Assigning Fuzzy Numbers to Typical Connections: Types of typical connections were defined: **TYPE-A(TA), TYPE-B(TB), TYPE-C(TC), TYPE-D(TD1/2/3), TYPE-E(TE)**, with their fuzzy triangular relationships shown in Fig 11.

Specifically: **TA (No Connection)** has a triangular fuzzy value of [0,0,0]; **TB (Weak Connection)** has a triangular fuzzy value of [0,0,0.5]; **TC (Connection)** has a triangular fuzzy value of [0.5,1,1.5]; Based on examples of typical connections, **TYPE-D(Close Connection)** is subdivided into TYPE-D1, TYPE-D2, and TYPE-D3, where the TFN for TYPE-D1 and TYPE-D2 are [1.5, 2, 2.5], and the TFN for TYPE-D3 is [1, 1.5, 2]; **TE (Highly Close Connection)** has a TFN of [2.5,3,3]. Except for TA, all typical connections are clearly defined with their fuzzy attributes.

(2) **Parameter Selection**

The accurate characterization of fuzzy path intensity depends not only on the construction of theoretical frameworks but also on appropriate parameter settings that reflect domain-specific network characteristics. In the specific application context of curriculum networks, parameter selection must balance theoretical rigor with practical operability. This study adopts an expert consensus-based parameter calibration method, ensuring that parameter settings align with both theoretical expectations and practical realities through iterative optimization. The parameter calibration process follows these principles: First, 3–4 typical node pairs are randomly selected for experimental evaluation; second, parameters are selected through group discussion, and the evaluation results are verified for acceptability until consensus is reached. While this approach incorporates certain subjective elements, it effectively integrates quantitative analysis with qualitative cognition, making it particularly suitable for complex systems rich in educational context such as curriculum networks. Based on this process, the following parameter settings were determined:

Intermediary strength coefficient *r* = 1.2: This parameter reflects the moderating role of intermediary nodes in influence propagation. Specifically, when two courses are closely connected, they can more effectively facilitate knowledge integration as intermediary nodes in each other’s influence paths. Intermediary node decay coefficient *ρ *= 1: This parameter characterizes the inhibitory effect of path length on influence intensity. *ρ *= 1 corresponds to harmonic series decay, indicating that influence continuously decreases with increasing intermediary nodes but never completely vanishes. This setting aligns with the cumulative effect in curriculum learning—even through multiple intermediate stages, the influence of foundational courses can still reach subsequent specialized courses. Optimal path weight coefficient *θ₁* = 1: The weight of the optimal path is normalized to 1, serving as the reference baseline for other paths’ influence. Secondary path influence coefficient *θ₂* = 0.2: This parameter characterizes the relative importance of multi-path influence between the same course pair in vocational education curriculum networks. The primary path represents the core regulatory mechanism of knowledge transmission, embodying critical dependencies and direct influence between courses; secondary paths constitute auxiliary regulatory mechanisms, providing supplementary channels for knowledge understanding and integration [54]. Secondary path decay coefficient *z* = 1: Adopting a power-law decay form *(1/k)* ensures that path influence decreases reasonably with ranking position, preventing excessive accumulation of low-intensity paths.

Furthermore, considering both computational efficiency and practical relevance, this study implements two path filtering constraints: ① paths with length greater than 8 are excluded, as such extended influence chains have limited practical significance in curriculum networks; ② paths with FIP values below 0.001 are omitted, given their negligible contribution to the network analysis.

(3) **Develop evaluation software**

To address issues such as the large number of course nodes, extended evaluation periods, and potential biases in understanding certain courses, this study developed a course network evaluation system based on Python. This system allows evaluators to simultaneously view detailed course information and previously evaluated results, aiming to enhance the efficiency and accuracy of the evaluation process.

**Fig 12:**
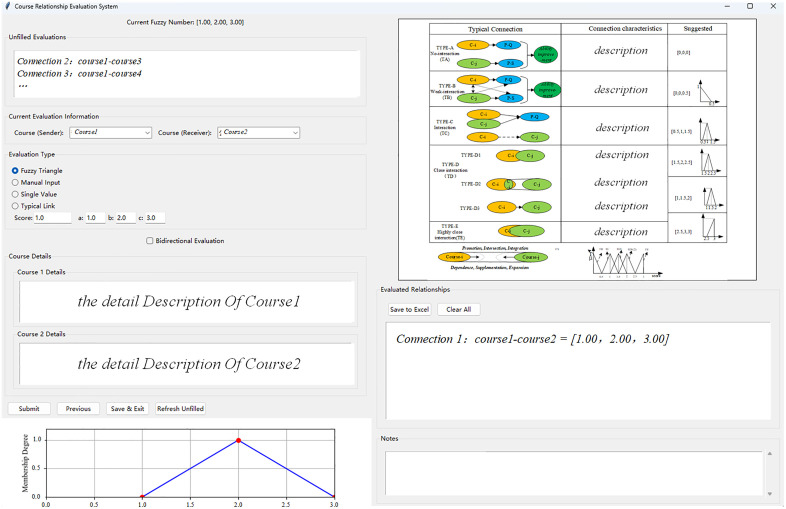
Fuzzy relationship evaluation system among courses.

Regarding the evaluation content, to address the fuzziness and uncertainty inherent in the evaluation process, the system offers multiple flexible evaluation methods:**①Selection of predefined typical connections**: Evaluators can directly select predefined types of typical connections (e.g., weak connection, close connection, etc.) from the system; **②Flexible dragging of fuzzy graphical elements**: The system supports adjustments to evaluation parameters by dragging fuzzy graphical elements, providing a more intuitive way to represent the fuzzy relationships between courses; ③**Direct input of fuzzy data:** In the later stages of the evaluation, assessors have gradually developed an experiential evaluation pattern and prefer to minimize software interactions. To address this, the approach of direct input of fuzzy data can be adopted. **④Direct input of rigid data**: For clearly defined non-fuzzy tendencies, evaluators can directly input specific numerical values. This diversified evaluation approach better accommodates the needs of different user groups, improving flexibility and user experience. The specific program interface design is shown in the Fig 12. The schematic diagram in the upper right corner illustrates typical connections among courses, designed to assist evaluators in consistently focusing on these connections during the assessment process, thereby ensuring evaluation consistency.

#### 5.2.2. Fuzzy Network construction of URTOMM.

A total of 39 courses were identified for URTOMM. After excluding ideological and political courses, 27 courses were evaluated in detail. The specific list of courses is as Table 5.

**Table 5:** Course nodes of URTOMM.

M1 Fundamentals of Computer Application	M10 Sign Language for Urban Rail Transit Customer Service	M19 Passenger Organization of Urban Rail Transit
M2 General English	M11 English for Urban Rail Transit Passenger Service	M20 Passenger Service of Urban Rail Transit
M3 Applied Mathematics	M12 Service Etiquette of Urban Rail Transit	M21 Train Operation Organization of Urban Rail Transit
M4 Electrical Engineering and Electronic Technology	M13 Driving of Electric Trains in Urban Rail Transit	M22 Operation Safety of Urban Rail Transit
M5 Introduction to Urban Rail Transit	M14 Psychology of Urban Rail Transit Operation Service	M23 Application of Electromechanical Equipment in Urban Rail Transit Stations
M6 Fundamentals and Practice of Management	M15 Dispatching and Commanding of Urban Rail Transit	M24 Comprehensive Training on Passenger First Aid
M7 Urban Rail Transit Communication and Signaling	M16 Specialized English for Urban Rail Transit	M25 Comprehensive Training on Passenger Service and Ticketing
M8 Fundamentals of Urban Rail Transit Vehicles	M17 Regulations on Urban Rail Transit Operation and Management	M26 Comprehensive Training on Station Affairs
M9 Urban Rail Transit Lines and Stations	M18 Automatic Fare Collection System and Ticket Handling of Urban Rail Transit	M27 Pre-job Training for Urban Rail Transit Operation

Based on the identified nodes, the curriculum network of URTOMM(CNURTOMM) was established, resulting $G_{URTOMM}=\left\{V,\tilde{A}\right\}$, where $V=\left\{M_{1},M_{2}....M_{27}\right\}$ denotes the set of nodes:

$\tilde{A}=\left(\begin{array}{ccc} {}*20c{\tilde{A}}_{11} & \cdots & {\tilde{A}}_{1\cdot 27}\\ \vdots & \ddots & \vdots \\ {\tilde{A}}_{27\cdot 1} & \cdots & {\tilde{A}}_{27\cdot 27} \end{array}\right)$ is the fuzzy adjacency matrix corresponding to nodes.

where ${\tilde{A}}_{ij}=\left[a_{ij},b_{ij},c_{ij}\right]$ represents the influence relationship from node *i* to node *j*.

### 5.3. Network analysis of CNURTOMM

#### 5.3.1. Fuzzy Degree Centrality of CNURTOMM.

**Fig 13:**
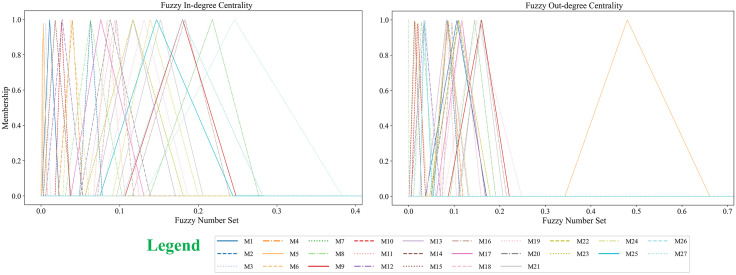
Fuzzy degree centrality of CNURTOMM.

**Fig 14:**
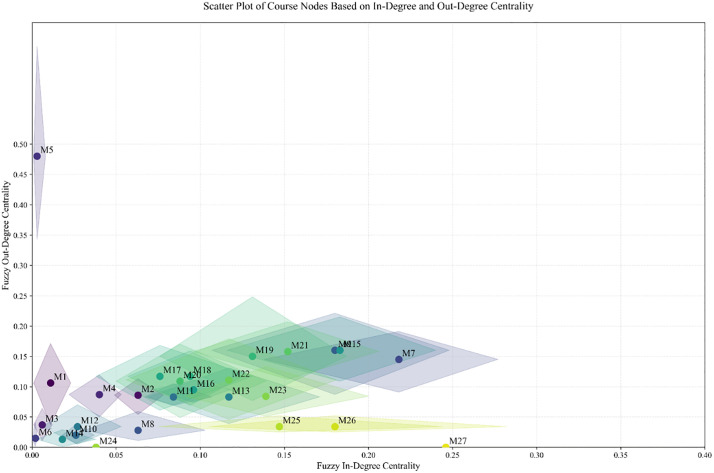
Fuzzy scatter plot of CNURTOMM based on fuzzy degree centrality.

Calculation of Fuzzy Degree Centrality Indexes based on Equations (18–21). **Figs 13**, **14, and Table 6** present the index results of fuzzy centrality, where the fuzzy out-degree and fuzzy in-degree centrality indexes are displayed in the form of TFN, and the overall situation is represented by quadrilaterals formed by fuzzy in-degree and fuzzy out-degree values. Each vertex of the quadrilateral represents the actual fuzzy value, providing an accurate depiction of the fuzzy characteristics of the nodes. The analysis results are as follows:

**Table 6:** The top six course nodes based on the fuzzy degree centrality ranking in CNURTOMM.

Course node	Fuzzy in-degree centrality (Value/rank)	Course node	Fuzzy out-degree centrality (Value/rank)	Course node	Fuzzy total-degree centrality (Value/rank)
M27	[0.131,0.246,0.383]/1	M5	[0.343,0.480,0.661]/1	M5	[0.343,0.482,0.668]/1
M7	[0.138,0.218,0.277]/2	M15	[0.109,0.160,0.215]/2	M7	[0.229,0.363,0.468]/2
M26	[0.105,0.180,0.282]/3	M21	[0.112,0.158,0.207]/3	M15	[0.226,0.343,0.455]/3
M15	[0.116,0.183,0.240]/4	M19	[0.077,0.150,0.248]/4	M9	[0.194,0.340,0.469]/4
M9	[0.107,0.180,0.248]/5	M9	[0.087,0.160,0.221]/5	M21	[0.208,0.310,0.413]/5
M25	[0.075,0.147,0.244]/6	M7	[0.091,0.145,0.191]/6	M19	[0.152,0.281,0.435]/6

(1) **In terms of fuzzy in-degree centrality index:**M27 has the highest fuzzy in-degree centrality index, which aligns with practical observations. As the final practical training course before job placement, its effects are significantly supported by preceding courses. Additionally, M7, M26, and M15 also exhibit high in-degrees, indicating that these courses are heavily influenced by other courses in the curriculum network and show strong dependence on preceding courses to achieve their objectives.
(2) **In terms of fuzzy out-degree centrality index:**M5 ranks first and far exceeds other courses, reflecting its critical supporting role in the overall curriculum system. This phenomenon is consistent with real-world course design: M5, as the comprehensive introductory course for the entire specialty, is typically scheduled in the first semester (1/6). This course provides a broad overview of general knowledge for the specialty and serves as an introductory course for students to deepen their understanding of subsequent courses. Additionally, M15, M21, and M19 also exhibit high fuzzy out-degree centrality index, indicating their strong influence on other courses and their roles as core nodes in the curriculum network.
(3) **Overall situation:** Fig 14 visually demonstrates the distribution of fuzzy in-degree and fuzzy out-degree centralities for each course node and their fuzzy characteristics. M5 has a high fuzzy out-degree and a low in-degree, indicating its clear precedence role in the curriculum system. M27, on the other hand, has no fuzzy in-degree and a relatively high fuzzy out-degree, with minimal fuzziness for both, highlighting its clear sequential order and key direct influence in the curriculum system. In practice, M5 is typically scheduled in the first semester, while M27 is placed in the final semester. Additionally, M7, M15, and M9 exhibit high fuzzy out-degrees, in-degrees, and fuzziness, suggesting their dominant positions in the curriculum network and their critical roles in connecting other courses.

#### 5.3.2. Fuzzy Betweenness Centrality of CNURTOMM.

**Fig 15:**
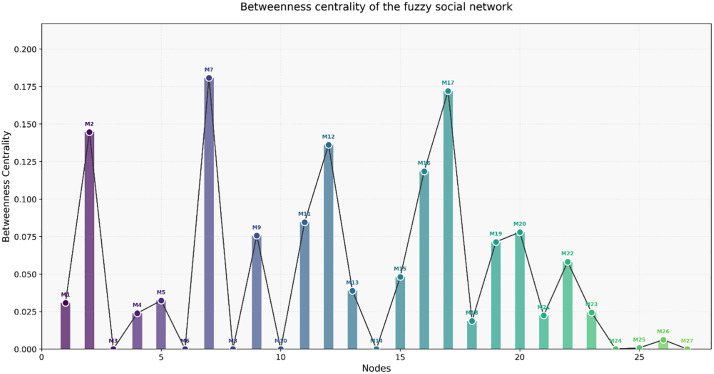
Fuzzy betweenness centrality of the CNURTOMM.

Calculation of Fuzzy Betweenness Centrality based on Equations (22–26). **Fig 15 and Table 7** present the fuzzy betweenness centrality analysis of the curriculum system, showing that M7 and M17 have the highest betweenness centrality indexes, indicating their roles as critical hubs in the entire curriculum network. Their teaching quality and content design directly influence the teaching effectiveness and learning efficiency of other related courses. By flexibly adjusting the teaching content of M7 and M17 (such as expanding knowledge modules and optimizing course sequence), more efficient integration of multiple courses can be achieved, thereby optimizing the overall teaching logic of the curriculum system and helping students build a clearer professional knowledge framework.

**Table 7:** The top six course nodes based on the fuzzy betweenness centrality ranking in CNURTOMM.

Course node	Value	Rank	Course node	Value	Rank
M7	0.181	1	M12	0.136	4
M17	0.172	2	M16	0.118	5
M2	0.145	3	M11	0.085	6

#### 5.3.3. Fuzzy Closeness Centrality of CNURTOMM.

**Fig 16:**
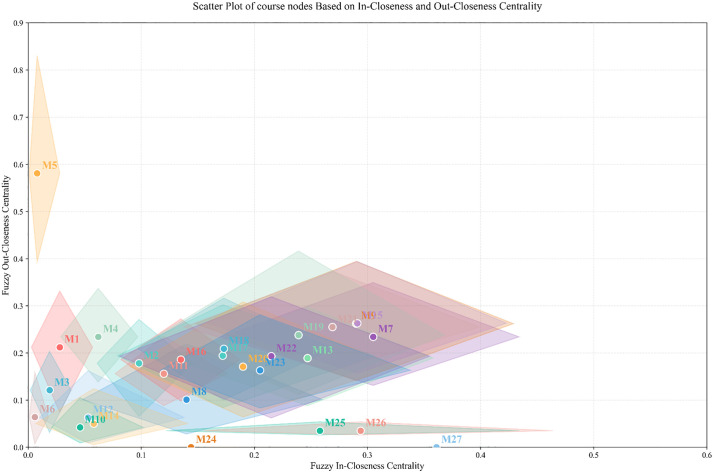
Fuzzy scatter plot of URTOMM based on fuzzy closeness centrality.

Calculation of Fuzzy Closeness Centrality Based on Equations (27–33). **Fig 16 and Table 8** present the results of fuzzy closeness centrality analysis, which reveals the influence relationships between courses from the perspective of the overall network. By analyzing fuzzy closeness centrality indexes, the position of courses in the curriculum system and their potential influence on other courses can be comprehensively understood.

**Table 8:** The top six course nodes based on the fuzzy closeness centrality ranking in CNURTOMM.

Course nodes	Fuzzy In-closeness centrality (Value/rank)	Course nodes	Fuzzy Out-closeness centrality (Value/rank)	Course nodes	Fuzzy Tot-closeness centrality (Value/rank)
M27	[0.185,0.361,0.559]/1	M5	[0.393,0.581,0.830]/1	M5	[0.394,0.589,0.858]/1
M26	[0.155,0.294,0.464]/2	M15	[0.157,0.263,0.394]/2	M15	[0.316,0.554,0.817]/2
M7	[0.171,0.305,0.434]/3	M9	[0.141,0.262,0.394]/3	M9	[0.289,0.552,0.823]/3
M15	[0.159,0.291,0.423]/4	M21	[0.162,0.255,0.373]/4	M7	[0.302,0.539,0.783]/4
M9	[0.148,0.290,0.429]/5	M19	[0.112,0.238,0.416]/5	M21	[0.300,0.524,0.772]/5
M25	[0.122,0.258,0.434]/6	M7	[0.131,0.234,0.349]/6	M19	[0.227,0.477,0.784]/6

A comparison of **Figs 13** and **16** reveals that the rankings of fuzzy closeness centrality and fuzzy degree centrality exhibit certain similarities, indicating a degree of alignment between direct influence and global influence. Fuzzy closeness centrality reflects broader fuzziness, which aligns with our objective understanding: the aggregation of more influencing factors results in greater fuzziness. Further analysis shows that the rankings of M15 and M9 improved in terms of fuzzy closeness centrality, suggesting an increase in their importance at the overall level. This finding highlights the following characteristics:

(1) **Key Roles from a Global Perspective**: These courses indirect influence through multiple paths in the broader curriculum network significantly increases their importance in global analysis.
(2) **Cumulative Effects in Fuzzy Networks**: These courses may carry more responsibilities in terms of knowledge association and information transfer, resulting in greater fuzziness. This suggests that in the optimization of the curriculum system, attention should be paid not only to directly core courses but also to these courses that exert influence through the global network.
(3) **Potential for Teaching Optimization**: Improving the rankings of M15 and M9 warrants further analysis of their specific course content and teaching design to determine how to better leverage their global importance, thereby enhancing the overall curriculum system.

Building on the aforementioned research, this study proposed a new scenario in the context of educational reform: as the coordination between courses strengthens, the influence of secondary paths becomes more pronounced. To simulate this change, while keeping other parameters unchanged, the importance weight of secondary paths is increased, and the attenuation coefficient of secondary paths is reduced, specifically adjusted to $\theta_{2}=0.8$, *z* = 0.5. The adjusted analysis results are presented in Table 8.

Table 9 presents the ranking of course nodes after increasing the importance of secondary paths. Compared with Table 7, certain changes in the rankings of course nodes are observed; however, most courses that ranked highly in Table 7 maintain their high rankings in Table 8, indicating the stability and effectiveness of core course identification. Notably, M21 and M9 show further improvement in their rankings for Fuzzy Total-Closeness Centrality, reflecting their potential importance within the course network and their supportive role in the overall curriculum system.

**Table 9:** The top six course nodes based on the fuzzy closeness centrality ranking in CNURTOMM. (In the scenario: the influence of secondary paths becomes more pronounced).

Course nodes	Fuzzy In-closeness centrality (Value/rank)	Course nodes	Fuzzy Out-closeness centrality (Value/rank)	Course nodes	Fuzzy Tot-closeness centrality (Value/rank)
M27	[1.047,3.007,5.883]/1	M5	[1.353,3.078,5.563]/1	M5	[1.357,3.102,5.650]/1
M26	[0.831,2.403,4.642]/2	M1	[0.526,1.838,3.635]/2	M27	[1.047,3.007,5.883]/2
M25	[0.687,2.002,3.924]/3	M4	[0.495,1.516,3.064]/3	M15	[1.135,2.966,5.650]/3
M22	[0.466,1.624,3.209]/4	M19	[0.539,1.401,2.785]/4	M21	[1.129,2.957,5.630]/4
M9	[0.565,1.572,2.981]/5	M15	[0.567,1.401,2.679]/5	M9	[1.116,2.936,5.589]/5
M15	[0.568,1.565,2.971]/6	M21	[0.578,1.382,2.654]/6	M7	[1.110,2.852,5.438]/6

### 5.4. Case comparison

For comparative analysis, this study selects the Courses Network of URTCSTM from the same institution as the research subject. CNURTCSTM identifies a total of 27 course nodes, with the fuzzy degree centrality analysis results presented in Table 10 and the fuzzy closeness centrality analysis results in Table 11.

**Table 10:** The top six course nodes based on the fuzzy degree centrality ranking in URTCSTM.

Course node	Fuzzy in-degree centrality (Value/rank)	Course node	Fuzzy out-degree centrality (Value/rank)	Course node	Fuzzy total-degree centrality (Value/rank)
T25: Pre-job Training for Urban Rail Transit(URT) Communication and Signaling	[0.140,0.219,0.303]/1	T5: Introduction to URT	[0.273,0.300,0.336]/1	T14: System Engineering Design of RTS	[0.239,0.343,0.474]/1
T23: Comprehensive Training in Mainline Signaling Equipment Maintenance for URT	[0.127,0.190,0.259]/2	T1: Fundamentals of Computer Applications	[0.125,0.180,0.301]/2	T18: Maintenance of Interlocking Systems in URT	[0.217,0.317,0.440]/2
T18: Maintenance of Interlocking Systems in URT	[0.124,0.179,0.268]/3	T10: Computer Network Technology	[0.109,0.192,0.288]/3	T5: Introduction to URT	[0.273,0.300,0.342]/3
T24: Comprehensive Training in Interlocking Control System Maintenance for URT	[0.127,0.182,0.246]/4	T14: System Engineering Design of RTS	[0.127,0.172,0.226]/4	T23: Comprehensive Training in Mainline Signaling Equipment Maintenance for URT	[0.169,0.267,0.377] 4
T14: System Engineering Design of Rail Transit Signaling(RTS)	[0.112,0.172,0.249]/5	T2: General English	[0.076,0.134,0.271]/5	T16: Maintenance of Basic Signaling Equipment in URT	[0.155,0.256,0.392]/5
T16: Maintenance of Basic Signaling Equipment in URT	[0.095,0.151,0.245]/6	T7: Fundamentals of Electronics and Process Training	[0.090,0.133,0.197]/6	T25: Pre-job Training for Urban Rail Transit Communication and Signaling	[0.159,0.258,0.360]/6

**Table 11:** The top six course nodes based on the fuzzy closeness centrality ranking in URTCSTM.

Course node	Fuzzy In-closeness centrality (Value/rank)	Course node	Fuzzy Out-closeness centrality (Value/rank)	Course node	Fuzzy Tot-closeness centrality (Value/rank)
T25	[0.211,0.355,0.528]/1	T5	[0.336,0.416,0.511]/1	T14	[0.353,0.576,0.844]/1
T24	[0.215,0.343,0.477]/2	T1	[0.193,0.297,0.487]/2	T18	[0.338,0.551,0.808]/2
T23	[0.206,0.335,0.482]/3	T10	[0.169,0.301,0.464]/3	T23	[0.291,0.510,0.767]/3
T18	[0.202,0.321,0.478]/4	T14	[0.169,0.261,0.381]/4	T16	[0.271,0.492,0.779]/4
T14	[0.184,0.315,0.463]/5	T2	[0.125,0.248,0.411]/5	T25	[0.273,0.491,0.755]/5
T16	[0.168,0.291,0.468]/6	T9	[0.149,0.232,0.378]/6	T24	[0.289,0.497,0.732]/6

Note: T9: PLC Technology and Applications; T24: Comprehensive Training in Interlocking Control System Maintenance for URT

(1) In the analysis of fuzzy degree centrality, the general foundational courses (e.g., T1, T2, T10) in URTCSTM exhibit higher centrality rankings compared to those in URTOMM. This finding suggests that in the URTCSTM curriculum system, general foundational courses play a more substantial supporting role for other specialized courses, likely due to the major’s higher reliance on fundamental theoretical knowledge.
(2) In the analysis of fuzzy closeness centrality, URTCSTM exhibits characteristics similar to URTOMM: high-degree nodes generally demonstrate higher closeness centrality. However, notable ranking discrepancies exist among certain nodes. For instance, while T5 remains ranked first in Fuzzy Out-closeness Centrality, its total degree ranking drops to 16th.This phenomenon indicates that, unlike the URTOMM, introductory courses in the URTCSTM do not hold a dominant position. Instead, certain core specialized courses (e.g., T14) have assumed more pivotal roles in the curriculum system.
(3) In URTOMM, courses ranked highly in fuzzy out-degree (closeness) centrality show significant overlap with those ranked highly in fuzzy total-degree (closeness) centrality. In contrast, in URTCSTM, courses ranked highly in fuzzy in-degree (closeness) centrality align more closely with those ranked highly in fuzzy total-degree (closeness) centrality. This phenomenon highlights a notable distinction between the two majors: URTOMM places greater emphasis on the development of practical work skills, with its curriculum design focused on real-world work scenarios, whereas URTCSTM places greater emphasis on the in-depth mastery of fundamental theories and scientific principles, with its curriculum design focusing more on the development of foundational theories and knowledge systems.
(4) A comparative analysis of the overall fuzzy centrality metrics for the two majors (Table 12) reveals that URTCSTM exhibits significantly higher values in Fuzzy Total-Degree Centrality, Fuzzy Betweenness Centrality, and Fuzzy Total-Closeness Centrality compared to URTOMM. This finding indicates that the inter-course connectivity in URTCSTM is notably stronger than in URTOMM. This phenomenon aligns with the practical characteristics of the majors: URTCSTM primarily targets engineering technical roles such as signal equipment and communication equipment, which require a highly systematic and interconnected knowledge structure; whereas URTOMM focuses on urban rail transit site management roles, emphasizing practical and flexible knowledge structures, resulting in relatively looser inter-course connections.

**Table 12:** Comparison of Network Indicators between CNURTCSTM and CNURTOMM.

Centrality index	CNURTOMM	CNURTCSTM
Fuzzy Total-Degree Centrality(Average)	[3.008,5.091,7.415]	[3.417,5.115,7.485]
Fuzzy Betweenness Centrality(Average)	0.0506	0.0677
Fuzzy Total-Closeness Centrality(Average)	[4.362,8.855,14.205]	[5.886,10.112,15.794]

### 5.5. Comparative analysis with existing methods

Sections 4.2.2 and 4.2.3 have theoretically demonstrated that the fuzzy centrality method proposed by Porreca et al. (2025) (hereafter referred to as the P-2025 method) can be viewed as a special case of the EDFSNAF framework proposed in this study. To validate the practical implications of this theoretical inference, this section presents an empirical comparison between the two methods. Using the same URTOMMCN, we systematically compare the EDFSNAF method and the P-2025 method in terms of parameter settings, computational results, and application scenarios, thereby revealing the substantial impact of different parameter configurations on fuzzy network analysis outcomes.

#### 5.5.1. Parameter configuration comparison.

The P-2025 method exhibits limitations in two critical aspects: First, it does not address how to establish and define fuzzy connection relationships across broader application domains, limiting its generalizability; second, it adopts a highly simplified parameter configuration. Specifically, the core assumptions of the P-2025 method can be mapped to the following specific parameter combinations within our framework:

(1) Intermediary strength coefficient *r* = 1.0: assumes influence maintains its original intensity during cross-node transmission, without considering the moderating effects of intermediary nodes; (2) Path decay coefficient ρ=0: path length has no inhibitory effect on influence intensity, i.e., no distance decay phenomenon exists; (3)Path weight coefficients *θ₁ = 1,θ₂ = 0:* considers only the optimal path between node pairs while completely ignoring contributions from secondary paths.This parameter configuration represents an extreme application of the “bottleneck principle”—assuming that inter-node influence relationships are entirely determined by a single strongest path, with the influence intensity of this path unaffected by distance. In contrast, the EDFSNAF method, through its parameterized design, systematically integrates three key mechanisms: the moderating effects of intermediary nodes, influence decay due to distance, and the cumulative effects of multiple paths, thereby more accurately assessing node importance in fuzzy networks.

#### 5.5.2. Comparison of centrality measure results.

**Fig 17:**
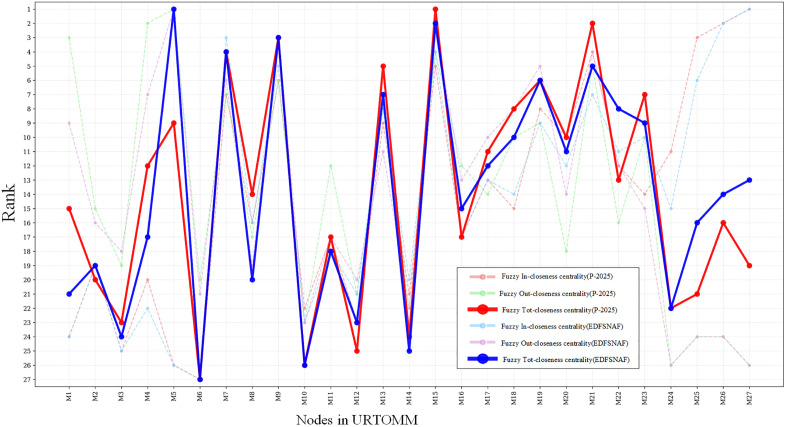
Comparison of fuzzy closeness centrality rankings between two methods.

Tables 13 and 14 present the computational results of the P-2025 method for betweenness centrality and closeness centrality, respectively, while Fig 17 illustrates the ranking differences in closeness centrality results between the two methods. The main findings include:

**Table 13:** Fuzzy betweenness centrality rankings in CNURTOMM (P-2025 method).

Course node	Value	Rank	Course node	Value	Rank
M7	0.399	1	M17	0.289	4
M9	0.381	2	M19	0.285	5
M15	0.345	3	M21	0.250	6

**Table 14:** The top six course nodes based on the fuzzy closeness centrality ranking in URTOMM (P-2025 method).

Course nodes	Fuzzy In-closeness centrality (Value/rank)	Course nodes	Fuzzy Out-closeness centrality (Value/rank)	Course nodes	Fuzzy Tot-closeness centrality (Value/rank)	ΔFuzzy Tot-closeness (P-2025 − EDFSNAF)
M27	[0.256,0.445,0.626]/1	M5	[0.366,0.511,0.704]/1	M15	[0.399,0.684,0.985]/1	[0.08,0.13,0.17]
M26	[0.257,0.417,0.581]/2	M4	[0.269,0.404,0.546]/2	M21	[0.400,0.684,0.984]/2	[0.10,0.16,0.21]
M25	[0.213,0.357,0.550]/3	M1	[0.227,0.392,0.550]/3	M9	[0.387,0.676,0.973]/3	[0.10,0.12,0.15]
M21	[0.187,0.357,0.485]/4	M15	[0.212,0.327,0.500]/4	M7	[0.388,0.661,0.953]/4	[0.09,0.12,0.17]
M15	[0.187,0.357,0.485]/5	M21	[0.213,0.327,0.499]/5	M13	[0.391,0.652,0.938]/5	[0.18,0.22,0.28]
M9	[0.181,0.353,0.479]/6	M9	[0.206,0.323,0.494]/6	M5	[0.372,0.539,0.789]/9	[-0.02,-0.05,-0.07]

(1) Betweenness Centrality Comparison: The analysis reveals that, except for M7 consistently ranking first in both methods, the top 6 bridging nodes are completely different. This significant discrepancy demonstrates the substantial impact of path intensity decay mechanisms on the assessment of node bridging roles. The EDFSNAF method, by incorporating distance decay and intermediary moderation, identifies key bridging nodes that better reflect courses serving as knowledge transfer hubs in actual teaching processes; In contrast, the P-2025 method tends to identify long paths composed of consecutive strong connections; however, in practical curriculum networks, such lengthy chain-like connections spanning multiple courses often lack pedagogical relevance and fail to capture the authentic relationships between courses. The consistent top ranking of M7 in both methods firmly establishes its robust bridging role and central position in the curriculum system.
(2) Closeness Centrality Comparison: In the EDFSNAF method, M5 ranks first in both fuzzy out-closeness centrality and total closeness centrality, a result that highly aligns with its functional positioning as an introductory professional course. However, in the P-2025 method, M5’s total closeness centrality ranking drops dramatically to 9th place. This dramatic shift reveals fundamental divergences in how the two methods conceptualize network structure:
  ① First, as an introductory course, M5’s network characteristics manifest as moderate-strength connections with multiple subsequent courses, forming a pattern of “broad radiation” influence. Under the EDFSNAF framework, these diversified propagation paths are fully captured through cumulative effect mechanisms—even when individual path strengths are not optimal, the synergistic effects of multiple paths still generate significant overall influence. In contrast, the P-2025 method focuses solely on the single strongest path between nodes, causing M5’s multi-path propagation advantages to be systematically overlooked.
  ② More critically, the P-2025 method’s failure to consider distance decay (*ρ* = 0) allows paths that should be filtered out to maintain unreasonably high influence. This design flaw produces dual negative effects: on one hand, other nodes can achieve influence comparable to direct connections through circuitous long paths (e.g., passing through 4–5 intermediary nodes), artificially elevating their closeness centrality rankings; on the other hand, the relative importance of nodes with genuine structural advantages (such as M5) becomes diluted. This “distance-independent” assumption violates fundamental principles of information propagation—in real-world networks, influence inevitably decays with increasing propagation distance.

The numerical differences presented in Tables 14 are precisely the result of these two mechanisms working in tandem. The EDFSNAF method, by introducing a distance decay coefficient and multi-path aggregation mechanisms, not only identifies M5’s central position but also appropriately suppresses the undue influence of long paths, providing an analytical framework that better conforms to real-world network behavior. This comparison clearly demonstrates that in fuzzy network analysis, appropriate path strength modeling plays a decisive role in accurately assessing node importance. It is particularly noteworthy that M15, M21, M9, and M7 consistently rank highly in both methods and under the “enhanced secondary path influence” scenario explored in Section 5.3.3. This robustness demonstrates that these courses not only feature strong direct connections but also hold critical positions within the multi-path propagation structure of the curriculum network, with their central importance being invariant to parameter configurations and method selection.

#### 5.5.3. Discussion on method applicability.

Based on the theoretical analysis and empirical comparison above, the two methods exhibit distinct complementary characteristics in fuzzy network analysis. It is particularly important to emphasize that the P-2025 method is actually a special case of the EDFSNAF framework under specific parameter configurations (*r* = 1, *ρ* = 0, *θ₁* = 1, *θ₂* = 0). This inclusion relationship not only validates the theoretical completeness of the EDFSNAF framework but also reveals the essence of differences in method applicability—they are not mutually independent analytical paradigms, but rather specific implementations of the same theoretical framework in different parameter spaces.

(1) Application Scenarios and Limitations of the P-2025 Method

The P-2025 method, grounded in the “bottleneck principle,” suits network systems where path selection follows strict optimization criteria, such as network flow allocation and shortest path planning. In these systems, node importance is primarily determined by single optimal paths, without significant multi-path synergistic effects or distance decay phenomena.

However, such scenarios fundamentally belong to deterministic network analysis. Despite parameter uncertainty, propagation mechanisms follow explicit physical or logical rules [57]. Within this framework, the P-2025 method’s core assumptions—“considering only optimal paths” and “ignoring distance decay”—are justifiable. Yet when applied to fuzzy social networks, these assumptions become critical limitations: they fail to capture the essential characteristics of human behavior and social interactions—uncertainty, diversity, and cumulativeness—which constitute the core value of FSNA.

(2) Application Scenarios and Advantages of the EDFSNAF Method

The EDFSNAF method, through path intensity decay and multi-path aggregation mechanisms, is particularly suited for analyzing complex social networks with inherent fuzziness. Its core advantages include: ① accurately identifying key nodes that exert influence through multiple paths; ② capturing subtle differences in network relationships and cumulative effects; ③ authentically reflects the inherent uncertainty of human behavior and the complex dynamics of social interactions, with this multi-path analytical perspective forming a crucial foundation for understanding system behavior.

(3) Method Selection Strategy Under a Unified Framework

The theoretical inclusion relationship revealed in this study establishes a systematic decision-making framework for method selection in practical applications. This framework maps different types of fuzzy networks to specific regions within the parameter space, enabling researchers to achieve precise alignment between methods and network characteristics through parameter adjustment.

Specifically, for deterministic network systems oriented toward path optimization, it is recommended to adopt distance decay coefficients approaching zero (ρ→0) and secondary path weights approaching zero ($\theta_{2}\to 0$). This parameter configuration converges the model to the non-decay optimal path principle advocated by the P-2025 method, suitable for application scenarios where influence propagation between nodes follows strict optimization criteria and contributions from secondary paths are negligible.

In stark contrast, when dealing with complex fuzzy networks centered on human behavioral patterns and social interaction mechanisms, it becomes essential to fully leverage distance decay effects (ρ >0) and multi-path aggregation mechanisms (*θ₂* > 0). The inherent characteristics of these networks—incompleteness of information transmission, cumulativeness of influence effects, and multidimensionality of relational structures—precisely constitute the distinctive advantages of the EDFSNAF framework.

The aforementioned parameter configuration strategies demonstrate that the EDFSNAF framework, through flexible parameter settings, can adapt to a wide range of application scenarios from deterministic networks to complex social networks. This unified analytical perspective based on parameter space offers valuable exploration for fuzzy network analysis: First, it attempts to transcend the binary opposition thinking of traditional methodology, exploring the transformation of discrete method selection into an optimization problem within continuous parameter space; second, it provides interdisciplinary researchers with preliminary parameter configuration references, helping to enhance the practical application value of the theoretical framework; third, it offers a theoretical starting point for constructing intelligent parameter-adaptive mechanisms, indicating a potential research direction for the field of fuzzy network analysis.

## 6. Conclusion and future work

In this paper, we propose an Extended Directed Fuzzy Social Network Analysis Framework (EDFSNAF) to provide methodological support for FSNA across diverse domains. The main theoretical contributions are two fold: (1) We innovatively introduce the concept of Typical Connections in EDFSNAF, proposing a domain-agnostic approach for defining fuzzy relationships. By establishing a standardized relationship description framework, it enables systematic representation of fuzzy relationships across different fields. (2) EDFSNAF comprehensively considers the variable requirements arising from node heterogeneity and network complexity, expanding the representational space for influence relationships in fuzzy networks. By redefining Fuzzy Intensity of Path (FIP) and introducing Total Fuzzy Intensity of Path (TFIP), our framework achieves a smooth transition from single optimal path analysis to multi-path aggregation analysis, providing a flexible parameterized framework for calculating fuzzy betweenness and closeness centrality.

To validate EDFSNAF’s effectiveness, we first tested the proposed FIP and TFIP using two illustrative examples, then conducted empirical analysis on China’s vocational education curriculum system. This domain offers unique advantages: theoretically, it combines human factor assessments (from teachers and industry experts) with structural complexity distinct from traditional interpersonal networks; practically, China’s ongoing vocational education reform urgently needs scientific methods for curriculum evaluation and resource optimization, making our framework directly applicable to real-world educational challenges. Specifically, we analyzed curriculum networks from two rail transit majors: Urban Rail Transit Operation and Management Major (URTOMM) and Urban Rail Transit Communication and Signaling Technology Major (URTCSTM) for empirical analysis. Case study results indicated that M5, M27, M26, M7, M9, and M15 as core courses in the URTOMM curriculum network, with their significant centrality metrics demonstrating their pivotal roles and influence within the network. Through comparatived analysis of the overall network metrics between URTOMM and URTCSTM, the study revealed that the two disciplines exhibit significantly differentiated characteristics in their network structures: The URTOMM curriculum network demonstrates a structure with introductory courses as the absolute core, while URTCSTM exhibits stronger supporting roles of fundamental courses. Furthermore, methodological comparison reveals that M5’s fuzzy tot-closeness centrality ranking drastically drops from 1st to 9th place under the P-2025 method, severely misaligning with practical experience. This deviation reflects the inherent limitations of considering only non-decay single optimal paths, highlighting the practical value of our parameterized approach. These findings not only validate EDFSNAF’s application value in uncovering latent structures and differences in professional curriculum systems but also confirm the framework’s effectiveness in addressing fuzzy relationship assessment in networks, providing reliable methodological support for FSNA.

**Limitations and Future Research** While this study attempts to overcome existing limitations in fuzzy network analysis, we acknowledge that a single paper cannot address all challenges comprehensively. We present four aspects of limitations and corresponding future research directions:

(1) Applicability Analysis

The EDFSNAF framework directly addresses two core challenges in existing FSNA: the difficulties in handling relationship fuzziness and network heterogeneity characteristics, which stem from the lack of universal relationship modeling paradigms and adaptive centrality measurement mechanisms. By innovatively introducing the concept of typical connections and constructing a parameterized centrality measurement system, this framework breaks through the rigid constraints of traditional methods and demonstrates the potential for differentiated network analysis within a unified theoretical architecture, offering a possible exploratory direction for cross-domain fuzzy network analysis.

However, the trade-off between universality and precision constitutes an inherent limitation of this framework, a tension that manifests across multiple levels of theoretical construction.

First, although the typical connection mechanism offers cross-domain applicability, it may not fully capture the specialized and distinctive node relationships specific to particular domains.

Second, the centrality measurement system constructed in this framework—while seeking more accurate descriptions than existing research—essentially represents a theoretical reduction of fuzzy social network phenomena. This reduction does not pursue perfect correspondence with reality but seeks a pragmatic balance between mathematical tractability and phenomenological complexity. As revealed in Sections 4.2.2 and 4.2.3, the definition of path intensity and choice of aggregation operators possess inherent non-uniqueness—different modeling strategies may be equally valid based on different theoretical assumptions. This theoretical pluralism precisely reflects the essential nature of fuzzy network analysis: when confronting social systems replete with uncertainty, any analytical framework can only provide effective approximations from specific perspectives rather than exact representations. The design philosophy of EDFSNAF acknowledges this epistemological stance, providing adaptive space for different theoretical perspectives and practical needs through parameterization mechanisms.

Third, while parameterized design enhances model flexibility, it simultaneously introduces new methodological challenges. Parameter selection inevitably involves subjective judgment. Although this study adopts an expert consensus-based calibration method, this approach itself acknowledges the impossibility of complete objectification. Furthermore, the interaction effects in multi-parameter systems exhibit high nonlinearity, causing the search for optimal parameter combinations to face combinatorial explosion. More fundamentally, the definition of “optimal” parameter combinations inherently depends on specific evaluation criteria and application contexts. This contextual dependency further weakens the framework’s capacity to provide universal theoretical guidance.

Nevertheless, these limitations should not be viewed as flaws but rather as manifestations of inherent challenges in fuzzy network analysis. The value of the EDFSNAF framework lies precisely in confronting these challenges and providing systematic solutions. While parameterized design increases complexity, it provides necessary adaptability for different scenarios; though the typical connection mechanism incurs abstraction losses, it enables cross-domain comparability; and while theoretical approximation is imperfect, it has proven effective in practice. More importantly, this framework makes these trade-offs transparent and systematic, enabling researchers to make informed choices based on specific needs—itself a significant methodological advancement.

Based on these insights, future research can deepen and extend this framework in three directions: ①Conduct systematic cross-domain validation through large-scale empirical studies to evaluate the framework’s applicability boundaries and construct domain-parameter mapping knowledge bases; ②Develop adaptive parameter optimization mechanisms by exploring machine learning-based automatic tuning methods to achieve intelligent parameter configuration; ③Advance domain-specific customization by developing specialized typical connection libraries and professional metrics while maintaining core framework stability, achieving organic unity between universality and specialization.

(2) Computational Complexity

The EDFSNAF framework inevitably introduces computational complexity challenges while pursuing analytical accuracy. Compared to traditional methods that only consider optimal paths, this framework requires enumerating and evaluating multiple possible paths between nodes through the introduction of path intensity decay mechanisms (parameters r and ρ) and multi-path aggregation mechanisms (parameter θ₂), resulting in non-linear growth of computational load with network scale and density.

For a network with *n* nodes, the complexity analysis reveals stark differences between approaches. Traditional optimal path-based methods (e.g., P-2025) exhibit O(*n³*) time complexity, while EDFSNAF reaches O (*n² × k × p*), where k denotes the average number of paths between nodes and p represents the path intensity computation cost. For fuzzy closeness centrality calculation with θ₂ > 0, the complexity becomes O (*n² × k × (p + log k*)) due to the sorting operations in the aggregation process. In dense networks, *k* may grow exponentially with respect to the maximum path length, potentially causing actual complexity to exceed traditional methods. This computational burden, while enabling more accurate capture of complex influence propagation characteristics in fuzzy networks, creates substantial bottlenecks for large-scale analysis. Our empirical results demonstrate adequate performance for networks up to 30 nodes, but larger networks face exponential growth in computation time, highlighting the urgent need for more efficient approximation algorithms.

It is worth noting that computational complexity challenges stem not only from the algorithm itself but also from the inherent characteristics of fuzzy network analysis. When constructing large-scale fuzzy networks, determining fuzzy relationships between nodes constitutes another critical bottleneck—obtaining fuzzy evaluations for *n²* potential connections requires substantial domain knowledge and human participation. Future research could explore machine learning-based relationship inference methods to infer global fuzzy relationships from local observations, or adopt hierarchical sampling strategies that prioritize evaluating connection strengths for key node pairs, thereby reducing problem scale at the network construction stage. Meanwhile, by combining approximation algorithms, parallel computing architectures, and adaptive parameter adjustment strategies, computational efficiency can be enhanced while maintaining theoretical analytical advantages, enabling EDFSNAF to effectively scale to FSNA scenarios with thousands of nodes or larger.

(3) Network-level Structural Metrics and Visualization Methods

While the averaged fuzzy centrality metrics employed in this study provide preliminary insights into overall network structural differences, such simple arithmetic averaging may obscure the distributional characteristics of centrality values across nodes. Future research should explore more sophisticated network-level metrics, such as fuzzy entropy measures and fuzzy clustering coefficients that account for triangular fuzzy numbers, to more comprehensively characterize the global properties of fuzzy networks.

Furthermore, the inability to visualize fuzzy network structures represents a common limitation of both this study and existing research. Future work should develop specialized fuzzy network visualization methods, including multidimensional visualization techniques, interactive visualization platforms, and innovative visual encoding schemes, to intuitively present the fuzzy characteristics and uncertainty of networks, thereby providing effective tools for deeper understanding of fuzzy network structures.

(4) Limitations in the Case Study

In the context of curriculum network analysis, to address the multidimensional and complex relationships among courses and to focus on the identification of core courses, this study adopted a unified metric for proximity relationships. This approach effectively reduced the complexity and computational difficulty of mapping connections from different dimensions into the same fuzzy space, but it may also result in the loss of key information. Future research should focus on the application and development of multi-dimensional vectors in fuzzy SNA to enhance the comprehensiveness and accuracy of the analysis.
